# Perceived psychosocial impacts of legalized same-sex marriage: A scoping review of sexual minority adults’ experiences

**DOI:** 10.1371/journal.pone.0249125

**Published:** 2021-05-06

**Authors:** Laurie A. Drabble, Angie R. Wootton, Cindy B. Veldhuis, Ellen D. B. Riggle, Sharon S. Rostosky, Pamela J. Lannutti, Kimberly F. Balsam, Tonda L. Hughes

**Affiliations:** 1 College of Health and Human Sciences, San José State University, San José, California, United States of America; 2 School of Social Welfare, University of California, Berkeley, California, United States of America; 3 School of Nursing, Columbia University, New York, New York, United States of America; 4 Department of Political Science and Gender and Women’s Studies, University of Kentucky, Lexington, Kentucky, United States of America; 5 Educational, Counseling and School Psychology, University of Kentucky, Lexington, Kentucky, United States of America; 6 Center for Human Sexuality Studies, Widener University, Chester, Pennsylvania, United States of America; 7 Department of Psychology, Palo Alto University, Palo Alto, California, United States of America; 8 School of Nursing & Department of Psychiatry, Columbia University Irving Medical Center, New York, New York, United States of America; University of Toronto, CANADA

## Abstract

A growing body of literature provides important insights into the meaning and impact of the right to marry a same-sex partner among sexual minority people. We conducted a scoping review to 1) identify and describe the psychosocial impacts of equal marriage rights among sexual minority adults, and 2) explore sexual minority women (SMW) perceptions of equal marriage rights and whether psychosocial impacts differ by sex. Using Arksey and O’Malley’s framework we reviewed peer-reviewed English-language publications from 2000 through 2019. We searched six databases (PubMed, PsycINFO, CINAHL, Web of Science, JSTOR, and Sociological Abstracts) to identify English language, peer-reviewed journal articles reporting findings from empirical studies with an explicit focus on the experiences and perceived impact of equal marriage rights among sexual minority adults. We found 59 studies that met our inclusion criteria. Studies identified positive psychosocial impacts of same-sex marriage (e.g., increased social acceptance, reduced stigma) across individual, interpersonal (dyad, family), community (sexual minority), and broader societal levels. Studies also found that, despite equal marriage rights, sexual minority stigma persists across these levels. Only a few studies examined differences by sex, and findings were mixed. Research to date has several limitations; for example, it disproportionately represents samples from the U.S. and White populations, and rarely examines differences by sexual or gender identity or other demographic characteristics. There is a need for additional research on the impact of equal marriage rights and same-sex marriage on the health and well-being of diverse sexual minorities across the globe.

## Introduction

Legalization of same-sex marriage represents one important step toward advancing equal rights for sexual and gender minorities. Over the past two decades same-sex marriage has become legally recognized in multiple countries around the world. Between 2003 and mid-2015, same-sex couples in the United States (U.S.) gained the right to marry in 37 of 50 states. This right was extended to all 50 states in June 2015, when the U.S. Supreme Court ruled in Obergefell v. Hodges that same-sex couples in all U.S. states had equal marriage rights. As of October 2019, same-sex couples had the right to marry in 30 countries and territories around the world [1].

National laws or policies that extend equal marriage rights to same-sex couples signal a reduction in structural stigma and have the potential to positively impact the health and well-being of sexual minorities. Structural stigma refers to norms and policies on societal, institutional and cultural levels that negatively impact the opportunities, access, and well-being of a particular group [2]. Forms of structural stigma that affect sexual minorities—such as restrictions on same-sex marriage—reflect and reinforce the social stigma against non-heterosexual people that occurs at individual, interpersonal, and community levels [3]. According to Hatzenbuehler and colleagues, structural stigma is an under-recognized contributor to health disparities among stigmatized populations [4–6], and reductions in structural stigma can improve health outcomes among sexual minorities [7, 8].

Marriage is a fundamental institution across societies and access to the right to marry can reduce sexual-minority stigma by integrating sexual minority people more fully into society [9]. Same-sex marriage also provides access to a wide range of tangible benefits and social opportunities associated with marriage [9, 10]. Despite the benefits of marriage rights, sexual minorities continue to experience stigma-related stressors, such as rejection from family or community, and discrimination in employment and other life spheres [11]. In addition, reactions to same-sex marriage appear to differ among sexual minorities and range from positive to ambivalent [11–13]. Extending marriage rights to same-sex couples remedies only one form of structural stigma. Although legalization of same-sex marriage represents a positive shift in the social and political landscape, the negative impact of social stigma may persist over time. For example, a recent Dutch study found that despite 20 years of equal marriage rights, sexual minority adolescents continue to show higher rates of substance use and lower levels of well-being than their heterosexual peers [14]. This study underscores the importance of understanding the complex impact of stigma at the structural, community, interpersonal, and individual levels.

### Impact on sexual minority health

A growing body of literature, using different methods from diverse countries where same-sex marriage has been debated or adopted, provides important insights into the impact of equal marriage rights on the health and well-being of sexual minority individuals. Research to date has consistently found that legal recognition of same-sex marriage has a positive impact on health outcomes among sexual and gender minority populations [15–20]. Studies in the U.S. have found evidence of reduced psychological distress and improved self-reported health among sexual minorities living in states with equal marriage rights compared to those living in states without such rights [5, 21–23]. One state-specific study also found improved health outcomes for sexual minority men after legalization of same-sex marriage [24]. Furthermore, sexual minorities living in states that adopted, or were voting on, legislation restricting marriage recognition to different-sex couples reported higher rates of alcohol use disorders and psychological distress compared to those living in states without such restrictions [5, 25–31]. Consistent with research in the U.S., findings from research in Australia on marriage restriction voting, found that sexual minorities living in jurisdictions where a majority of residents voted in support of same-sex marriage reported better overall health, mental health, and life satisfaction than sexual minorities in locales that did not support same-sex marriage rights [32].

Although existing literature reviews have documented positive impacts of equal marriage rights on physical and mental health outcomes among sexual minority individuals [15–20], to our knowledge no reviews have conducted a nuanced exploration of the individual, interpersonal, and community impacts of legalized same-sex marriage. An emerging body of quantitative and qualitative literature affords a timely opportunity to examine a wide range of psychosocial impacts of equal marriage rights. Understanding these impacts is important to guide and interpret future research about the potential protective health effects of same-sex marriage.

### Potential differences between SMW and SMM

Given the dearth of research focusing on the health and well-being of sexual minority women (SMW), especially compared to the sizable body of research on sexual minority men (SMM) [33, 34], there is a need to explore whether the emerging literature on same-sex marriage provides insights about potential differences in psychosocial impacts between SMW and SMM. Recent research underscores the importance of considering SMW’s perspectives and experiences related to same-sex marriage. For example, gendered social norms play out differently for women and men in same-sex and different-sex marriages, and interpersonal dynamics and behaviors, including those related to coping with stress, are influenced by gender socialization [35]. However, there is little research about how societal-level gender norms and gendered social constructions of marriage may be reflected in SMW’s perceptions of same-sex marriage. Structural sexism (e.g., gendered power and resource inequality at societal and institutional levels) differentially impacts women’s and men’s health [36], and may also contribute to sex differences in experiences and impacts of same-sex marriage. For example, research from the U.S. suggests that same-sex marriage rights may improve health outcomes and access to healthcare for SMM, but evidence is less robust for SMW [37–39]. Differences in health outcomes appear to be at least partially explained by lower socioeconomic status (income, employment status, perceived financial strain) among SMW compared to SMM [40]. Further, other psychosocial factors may contribute to differential experiences of legalized same-sex marriage. For example, a study of older sexual minority adults in states with equal marriage rights found that married SMW experienced more LGBTQ (lesbian, gay, bisexual, transgender, queer) microaggressions than single SMW, but no differences by relationship status were noted among SMM [41]. Mean number of microaggressions experienced by SMW in partnered unmarried relationships fell between, but were not significantly different from, that of married and single SMW.

### Theoretical framework

**Fig 1:**
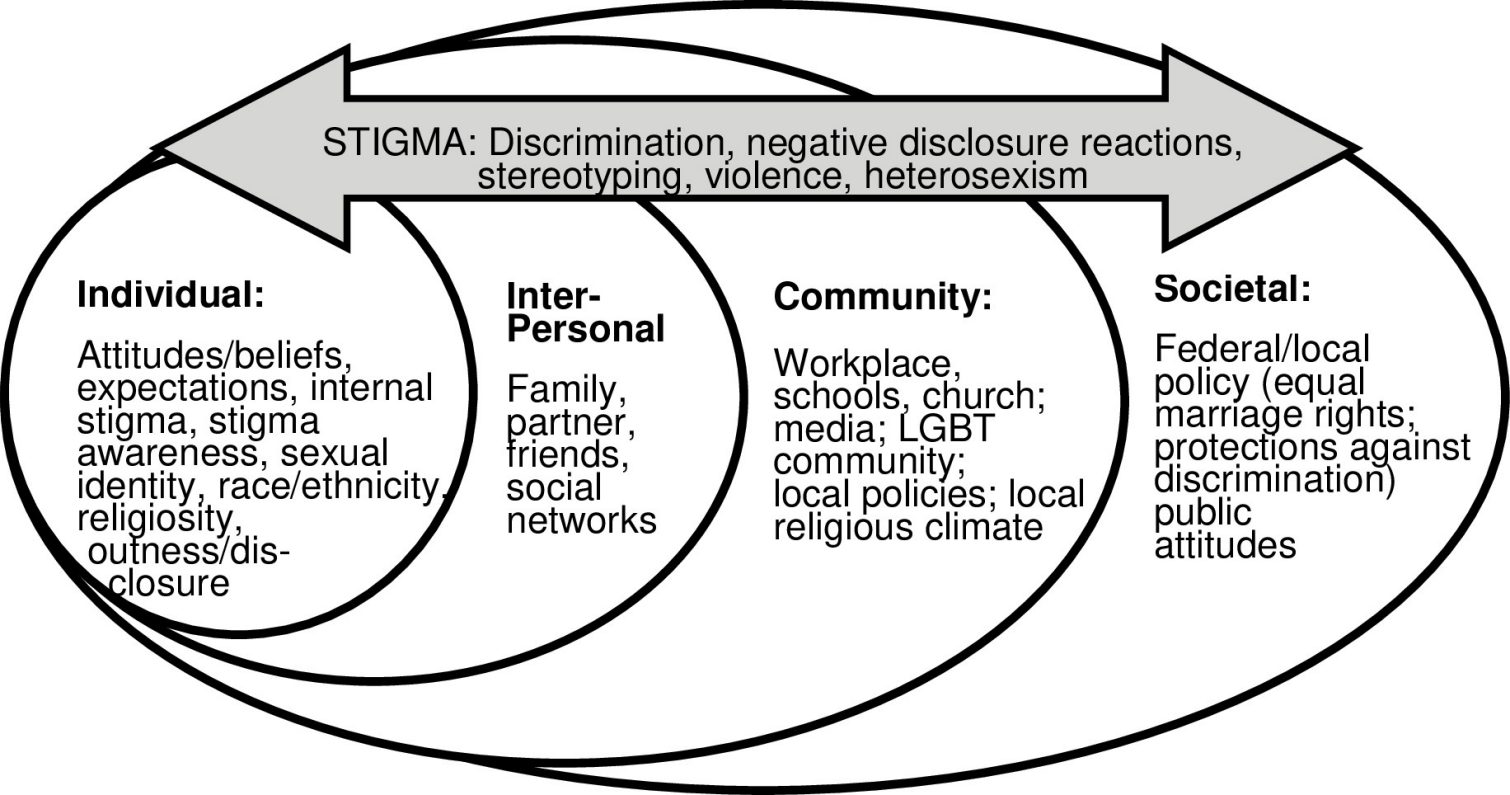
Social-ecological model: Impact of equal marriage rights among sexual minority adults.

Social-ecological and stigma theoretical perspectives were used as the framework for organizing literature in this review (See Fig 1). Stigma occurs and is experienced by sexual minorities at individual, interpersonal, and structural levels, which mirror the levels of focus within the social-ecological framework [6, 42]. Consequently, changes such as extending equal marriage rights to same-sex couples may influence sexual minorities’ experiences of stigma across all of these levels [43]. Gaining access to the institution of marriage is distinct from marital status (or being married) and likely impacts sexual minority adults across individual, interpersonal, and community contexts [44], regardless of relationship status.

From a social-ecological perspective, individual and interpersonal processes can amplify or weaken the impact of structural level policies, such as equal marriage rights, on sexual minority individuals’ health and well-being [43, 45, 46]. For example, on an individual level, experiences and perceptions of equal marriage rights may influence stigma-related processes such as internalized heterosexism, comfort with disclosure, and centrality of sexual identity [47]. Interpersonal and community level interactions may trigger stigma-related processes such as prejudice concerns, vigilance, or mistrust. Such processes may in turn, influence the impact of social policy change on sexual minority stress and well-being [48–50].

The impact of equal marriage rights among sexual minority individuals may also be influenced by other social and political factors such as state- or regional-level social climate [50–52], or inconsistency among other policy protections against discrimination (e.g., in housing or public accommodations) [11, 50]. Sociopolitical uncertainty may continue long after the right to marry is extended to same-sex couples [53, 54]. Monk and Ogolsky [44] define political uncertainty as a state of “having doubts about legal recognition bestowed on individuals and families by outside systems; being unsure about social acceptance of marginalized relationships; being unsure about how ‘traditional’ social norms and roles pertain to marginalized relationships or how alternative scripts might unfold” (p. 2).

### Current study

The overall aim of this scoping review was to identify and summarize existing literature on psychosocial impacts of equal marriage rights among sexual minority adults. Specific objectives were to: 1) identify and describe the psychosocial impacts of equal marriage rights on sexual minority adults; and 2) explore SMW-specific perceptions of equal marriage rights and whether psychosocial impacts differ for SMM and SMW.

## Methods

### Study design

We used a scoping review approach, as it is well-suited for aims designed to provide a descriptive overview of a large and diverse body of literature [55]. Scoping reviews have become a widely used approach for synthesizing research evidence, particularly in health-related fields [55]. Scoping reviews summarize the range of research, identify key characteristics or factors related to concepts, and identify knowledge gaps in particular areas of study [56, 57]. By contrast, systematic reviews are more narrowly focused on creating a critically appraised synthesized answer to a particular question pertinent to clinical practice or policy making [57]. We aimed to characterize and summarize research related to psychosocial impacts of equal marriage rights and same-sex marriage, including potential gaps in research specific to SMW. Following Arksey and O’Malley [56], the review was conducted using the following steps: 1) identifying the research question, 2) identifying relevant studies, 3) selecting studies, 4) charting the data, and 5) collating, summarizing and reporting results. Because this is a scoping review, it was not registered with PROSPERO, an international registry for systematic reviews.

### Selection method

The authors used standard procedures for conducting scoping reviews, including following PRISMA guidelines [58]. Articles that report findings from empirical studies with an explicit focus on the psychosocial impacts of equal marriage rights and same-sex marriage on sexual minority adults are included in this review. All database searches were limited to studies in English language journals published from 2000 through 2019 (our most recent search was executed in June 2020). This time frame reflects the two decades since laws regarding same-sex marriage began to change in various countries or jurisdictions within countries. Literature review articles and commentaries were excluded. To ensure that sources had been vetted for scientific quality by experts, only articles in peer-reviewed journals were included; books and research in the grey literature (e.g., theses, dissertations, and reports) were excluded. There was no restriction on study location. A librarian searched PubMed, PsycINFO, CINAHL (Cumulative Index to Nursing and Allied Health Literature), Web of Science, JSTOR, and Sociological Abstracts databases using combinations of key search terms. Following is an example of the search terms used in CINAHL database searches: ((TI "marriage recognition" OR AB "marriage recognition") OR (TI marriage OR AB marriage) OR (TI same-sex OR AB same-sex) OR (TI "same sex" OR AB "same sex")) AND ((TI LGBT OR AB LGBT) OR (TI gay OR AB gay) OR (TI lesbian OR AB lesbian) OR (TI bisexual OR AB bisexual) OR (TI transgender OR AB transgender) OR (TI Obergefell OR AB Obergefell) OR (TI "sexual minorities" OR AB "sexual minorities))

Articles were selected in two stages of review. In stage one, the first author and librarian independently screened titles and abstracts for inclusion or exclusion using eligibility criteria. We excluded articles focused solely on the impact of relationship status on health outcomes, satisfaction or dynamics within marriage relationships, or the process of getting married (e.g., choices of who to invite, type of ceremony), or other topics that did not pertain directly to the research aims. For example, a study about the impact of *getting* married that also included themes pertaining to *the impact or meaning* of equal marriage rights was included in the full review. The first author and a librarian met to review and resolve differences and, in cases where relevance was ambiguous, articles underwent a full-text review (in stage 2). Table 1 summarizes exclusion categories used in the title and abstract reviews.

**Table 1:** Exclusion categories used for title and abstract review.

Exclusion Category	Description (if applicable)
Not written in English	------
Not peer-reviewed	Conference proceedings, books and book chapters, magazine or news articles, theses/dissertations, reports and unpublished grey literature
Not an empirical study	Commentaries and editorials.
Review articles	Articles that did not present original research (e.g., systematic reviews, meta-analyses).
Not focused on adults	Articles focused on children or adolescents only
Not relevant	Articles that did not focus on psychosocial impacts of marriage legalization (e.g., legal analyses of marriage policies, dynamics in married relationships).
No sexual or gender minority (SGM) focus	Articles that did not sample SGM individuals or that did not focus on impacts in relation to sexual and gender minorities

**Fig 2:**
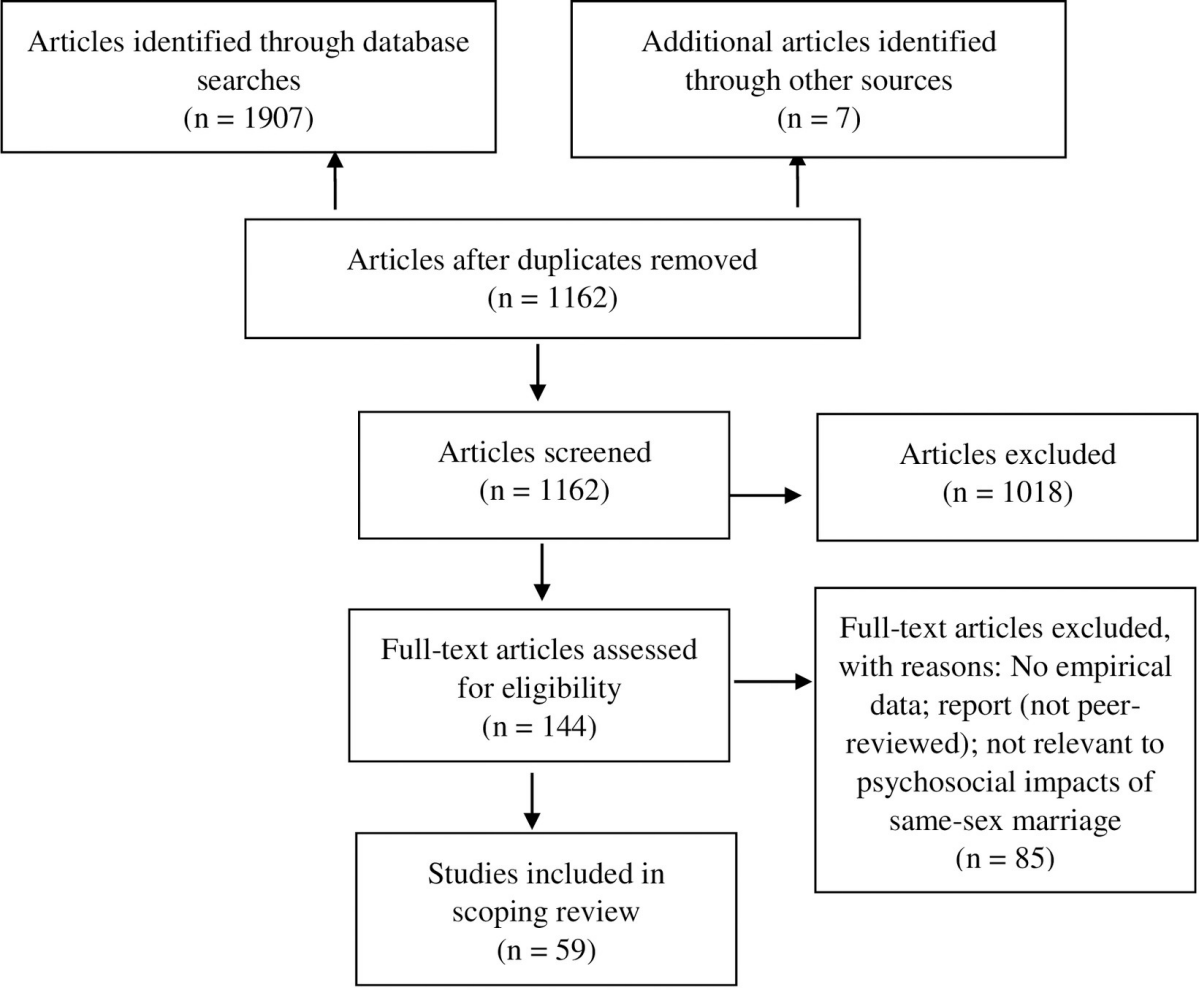
Studies identified and included in the literature review.

In stage two, articles not excluded in stage one were retrieved for full-text review. Each article was independently reviewed by two authors to assess study relevance. Discrepancies related to inclusion were few (less than 10%) and resolved through discussion and consensus-building among the first four authors. This process resulted in an analytic sample of 59 articles (see Fig 2).

Table 2 provides an overview of characteristics of the studies included in this scoping review. Most were qualitative and most aggregated SMW and SMM in analyses. Only 14 studies explored differences in impact for SMW and SMM, or separately examined the specific perceptions and experiences of SMW. Although search terms were inclusive of transgender individuals, samples in the studies we reviewed rarely included or focused explicitly on experiences of transgender or gender nonbinary identified individuals. In studies that explicitly included transgender and nonbinary individuals, sample sizes were rarely large enough to permit examination of differences based on gender identity (e.g., survey samples with 2–3% representation of nonbinary or transgender individuals) [44, 59–63]. Other studies recruiting sexual minorities may have included transgender and nonbinary individuals (who also identified as sexual minorities), but did not assess gender identity. Among studies in which participant race/ethnicity was reported, most included samples that were majority White.

**Table 2:** Summary of study characteristics (N = 59).

*Study Characteristics*		Count (%)
Study Design		
	Qualitative	31 (53)
	Quantitative	20 (34)
	Mixed methods	6 (10)
	Other (policy or case law analyses)	2 (3)
Study Location		
	U.S.	43 (73)
	Canada	2 (3)
	Europe	7 (12)
	Australia or New Zealand	3 (5)
	Multiple countries	4 (7)
Sample Composition–Sex/Gender		
	Only women	8 (14)
	Only men	3 (5)
	Mixed sex and gender	32 (54)
	Mixed sex and gender (transgender and nonbinary measured separately).	8 (14)
	Not reported	8 (14)
Sample Composition–Included Participants of Color %		
	75% or more	2 (3)
	50 to 74%	3 (5)
	25–49%	6 (10)
	Less than 25%	24 (41)
	Not reported	24 (41)
Analyses of Sub-Groups*		
	Sexual minority women	13 (22)
	SMW or SMM of color	5 (8)

* Not mutually exclusive, classifications do not equal 100%.

Studies of the impact of legalized marriage on physical health were not excluded in the original search parameters; however, physical health has been addressed in prior reviews [15–20]. Further, because our research questions focused on psychosocial factors, we excluded studies on physical health unless they also addressed individual, interpersonal, or community psychosocial impacts of same-sex marriage legalization. Studies that focused on physical health impacts or access to health insurance were used only in the introduction.

Civil union was not explicitly included as a search parameter, but articles focusing on civil unions were captured in our search. Although civil unions are not equivalent to marriage, they often confer similar substantive legal rights. We included articles about civil union that explicitly pertained to our research question, such as a study that examined perceived stigma and discrimination before and after implementation of civil union legislation in one U.S. state [64], and excluded articles that did not (e.g., a study of relationship quality or longevity among same-sex couples in civil unions) [65].

A majority of the studies were conducted in the U.S. Of the 43 U.S. studies, 20 sampled from a single state, 10 included participants from multiple states, 12 used a national sample, and one had no human subjects (secondary analysis of legal cases). Of those sampling a single state, all focused on the impact of changes (or proposed changes) in same-sex marriage policy: 10 focused on Massachusetts (the first state in the U.S. to legalize same-sex marriage), two focused on Iowa, two on Vermont, and two on California. One article each included study participants from Nebraska, Oregon, Illinois, and a small (unnamed) non-metropolitan town in the Midwest.

### Analysis method

We created a data extraction form to ensure consistency across team members in extracting key study information and characteristics including study design (e.g., quantitative, qualitative, or mixed method), location (e.g., country and/or region), sample (e.g., whether the study included or excluded SMW or SMM, assessed and reported race/ethnicity), and key results. Articles were also classified based on findings related to level of impact (e.g., individual, couple, family, community, or broader social attitudes toward LGBTQ+ individuals; see S1 Table). A final category on significance/implications allowed reviewers to further identify and comment on major themes and relevance to the current review. Themes were then identified and organized using stigma and social-ecological frameworks.

## Results

### Aim 1: Psychosocial impacts of same-sex marriage rights

#### Individual level impacts

Although most studies about the impact of equal marriage rights have been conducted with couples or individuals in committed or married relationships, 15 studies in this review included sexual minority adults across relationship statuses. In general, studies examining the impact of equal marriage rights among sexual minorities suggest that equal access to marriage has a positive impact on perceptions of social acceptance and social inclusion regardless of relationship status [47, 63, 66, 67]. For example, Riggle and colleagues [47] examined perceptions of sexual minority individuals in the U.S. during the period in which same-sex couples had equal marriage rights in some, but not all, U.S. states. Sexual minorities who resided in states with equal marriage rights reported less identity concealment, vigilance, and isolation than their peers in states without equal marriage rights. Similarly, using data from the longitudinal Nurses’ Health Study in the U.S., Charlton and colleagues [68] examined potential positive impacts of equal marriage rights on sexual identity disclosure. They found that participants living in states with any form of legal recognition of same-sex relationships (inclusive of marriage, civil unions, or domestic partnerships) were 30% more likely than those is states without legal recognition to consistently disclose a sexual minority identity across survey waves [68].

Researchers have documented ambivalence among sexual minority adults regarding the institution of marriage and whether same-sex marriage would impact other forms of structural or interpersonal stigma. Sexual minority participants in several studies expressed concern about continued interpersonal stigma based on sexual or gender identity, the limitations of marriage as a vehicle for providing benefits and protections for economically marginalized LGBTQ+ individuals, and the possibility that an increased focus on marriage would contribute to devaluing unmarried same-sex relationships [12, 13, 62, 69, 70]. Studies also documented concerns about marriage being inherently linked to heteronormative expectations and about assimilation to heterosexist cultural norms [60, 69, 71]. These concerns were summarized by Hull [69]: “The fact that LGBTQ respondents favor marriage more in principle (as a right) than in practice (as an actual social institution) suggests that marriage holds multiple meanings for them” (p. 1360).

Five studies explicitly examined racial/ethnic minority identities as a factor in individuals’ perceptions of same-sex marriage; one qualitative study focused exclusively on Black individuals in the U.S. [72] and the other four examined differences by race/ethnicity [64, 66, 67, 73]. McGuffy [72] conducted in-depth interviews with 102 Black LGBT individuals about their perceptions of marriage as a civil rights issue before and after same-sex marriage was recognized nationally in the U.S. The study found that intersecting identities and experiences of discrimination related to racism, homophobia, and transphobia influenced personal views of marriage. For example, although most participants were supportive of equal marriage rights as a public good, many felt that the emphasis on marriage in social movement efforts overlooked other important issues, such as racism, economic injustice, and transgender marginalization.

The four other studies examining racial/ethnic differences in perceptions about whether equal marriage rights facilitated inclusion or reduced interpersonal stigma yielded mixed results. One found that residing in states with equal marriage rights was associated with greater feelings of acceptance among sexual minorities; however, White sexual minorities reported greater feelings of inclusion than participants of color [66]. By contrast, in a quasi-experiment in which SMW in a midwestern state were interviewed pre- or post- passage of civil union legislation, those interviewed after the legislation reported lower levels of stigma consciousness and perceived discrimination than those interviewed before the legislation; however, effects were stronger among SMW of color than among White SMW [64]. In a study of unmarried men in same-sex male couples, Hispanic/Latino men were more likely than non-Latino White participants to report perceived gains in social inclusion after equal marriage rights were extended to all U.S. states [67]. However, men who reported higher levels of minority stress (enacted and anticipated stigma as well as internalized homophobia) were less likely to show improvement in perceptions of social inclusion. Lee [73], using data from a national Social Justice Sexuality Project survey, found no statistical differences in Black, White and Latinx sexual minorities’ perceptions that equal marriage rights for same-sex couples had a moderate to major impact on their lives. In analyses restricted to Black participants, individuals with higher level of sexual minority identity salience reported significantly higher importance of equal marriage rights. Lee suggests that same-sex marriage was perceived by many study participants as a tool to gain greater acceptance in the Black community because being married is a valued social status.

#### Couple level impacts

We identified 15 studies that focused on couples as the unit of analysis. Findings from studies of the extension of equal marriage rights in U.S. states suggest positive impacts among same-sex couples, including access to financial and legal benefits as well as interpersonal validation, such as perceptions of being viewed as a “real” couple and increased social inclusion [12, 59, 63, 74, 75]. Furthermore, couples in several studies described the potential positive impacts of legal recognition of their relationship on their ability to make joint decisions about life issues, such as having children and medical care [75]. Couples also described having a greater sense of security associated with financial (e.g., taxes, healthcare) and legal (e.g., hospital visitation) benefits and reduced stress in areas such as travel and immigration [75]. Collectively, these findings suggest that marriage rights were perceived to imbue individuals in same-sex relationships with a sense of greater security, stability, and safety due to the legal recognition and social legitimization of same-sex couples. Although equal marriage rights were perceived as an important milestone in obtaining civil rights and reducing institutional discrimination, concerns about and experiences of interpersonal stigma persisted [76–78]. The social context of legal same-sex marriage may create stress for couples who elect to not marry. For example, in a study of 27 committed, unmarried same-sex couples interviewed after the U.S. Supreme Court decision on Obergefell, couples who chose not to marry described feeling that their relationships were less supported and perceived as less committed [79].

Reports from the CUPPLES study, a national longitudinal study of same-sex couples in the U.S. from 2001 to 2014, provided a unique opportunity to examine the impact of different forms of legal recognition of same-sex relationships. In wave three of the study during 2013–2014, open-ended qualitative questions were added to explore how individuals in long-term committed partnerships perceived the extension of equal marriage rights in many U.S. states. Themes included awe about the historic achievement of a long-awaited civil rights goal, celebration and elation, and affirmation of minority sexual identity and relationships, but also fears of backlash against sexual minority rights [80]. Some individuals who divorced after institutionalization of the right to same-sex marriage reported shame, guilt, and disappointment—given that they and others had fought so hard for equal marriage rights [81].

Studies outside the U.S. have also found evidence of positive impacts of legal recognition of same-sex couple relationships (e.g., increased social recognition and social support), as well as potential concerns [82–86]. For example, in a study of couples from the first cohort of same-sex couples to legally marry in Canada, participants described marriage as providing them with language to describe their partner that was more socially understood and helping to decrease homophobic attitudes among the people around them [83]. Some couples said they could fully participate in society and that marriage normalized their lives and allowed them to “live more publicly.” Couples also discussed the safety, security, and increased commitment that came from marriage, and some felt that marriage opened up previously unavailable or unimagined opportunities, such as becoming parents. However, some participants noted that their marriage caused disjuncture in relationships with their family of origin, as marriage made the relationship feel too real to family members and made their sexual identities more publicly visible.

#### Family level impacts

Seventeen studies examined the impact of equal marriage rights on sexual minority individuals’ or couples’ relationships with their families of origin. Although these studies predominately used cross-sectional survey designs, one longitudinal study included individuals in both different-sex and same-sex relationships before and after the U.S. Supreme Court decision that extended marriage rights to all states [44]. This study found that support from family members increased following national legalization of same-sex marriage [44]. A cross-sectional online survey of 556 individuals with same-sex partners in Massachusetts (the first U.S. state to extend equal marriage rights to same-sex couples), found that greater family support and acceptance of same-sex couples who married was associated with a stronger overall sense of social acceptance [66].

Other cross-sectional surveys found mixed perceptions of family support and feelings of social acceptance. For example, a study of 357 participants in long-term same-sex relationships found that perceived social support from family did not vary by state-level marriage rights or marital status [47]. However, living in a state with same-sex marriage rights was associated with feeling less isolated. The finding of no differences in perceived support might be partly explained by the fact that the sample included only couples in long-term relationships; older, long-term couples may rely less on support from their family of origin than younger couples [12].

In studies (n = 6) that included dyadic interviews with same-sex married couples [74, 79, 85, 87–89], participants described a wide range of family members’ reactions to their marriage. These reactions, which emerged after same-sex marriage legalization, were typically described by couples as profoundly impactful. Couples who perceived increased family support and acceptance described these changes as triumphant [85], transformative [88], and validating [74, 87]. Conversely, some same-sex couples reported feeling hurt and betrayed when familial reactions were negative or when reactions among family members were divided [85, 87, 89]. Findings from these and other studies suggest that if certain family members were accepting or rejecting prior to marriage, they tended to remain so after equal marriage rights and/or the couple’s marriage [61, 74, 90, 91]. In some cases, family members were perceived as tolerating the same-sex *relationship* but disapproving of same-sex *marriage* [85, 90].

Findings from studies of married sexual minority people suggest that family (especially parental) disapproval was a challenge in the decision to get married [92], possibly because disclosure of marriage plans by same-sex couples frequently disrupted family “privacy rules” and long-time patterns of sexual identity concealment within families or social networks [87]. In a few studies, same-sex partners perceived that their marriage gave their relationship more legitimacy in the eyes of some family members, leading to increased support and inclusion [61, 66, 89–91]. Further, findings from two studies suggested that participating in same-sex weddings gave family members the opportunity to demonstrate support and solidarity [87, 93].

Two qualitative studies collected data from family members of same-sex couples. In one, heterosexual siblings (all of whom were in different-sex marriages) described a range of reactions to marriage equality—from support for equal marriage rights to disapproval [80]. The other study interviewed sexual minority migrants to sexual minority friendly countries in Europe who were married and/or raising children with a same-sex partner, and these migrant’s parents who lived in Central and Eastern European countries that prohibited same-sex marriage. Parents found it difficult to accept their adult child’s same-sex marriage, but the presence of grandchildren helped to facilitate acceptance [94].

#### Community level impacts

Twelve studies in this review examined the community-level impacts of same-sex marriage. These studies focused on community level impacts from two perspectives: impacts of equal marriage rights on LGBTQ+ communities, and the impacts of equal marriage rights on LGBTQ+ individuals’ interactions with their local communities or extended social networks.

*LGBTQ+ communities*. A prominent theme among these studies was that marriage is beneficial to LGBTQ+ communities because it provides greater protection, recognition, and acceptance of sexual minorities, their families, and their relationships—even beyond the immediate impact on any individual and their relationship or marriage [12, 62, 89, 95]. Despite these perceived benefits, studies have found that some sexual minority adults view marriage as potentially harmful to LGBTQ+ communities because of concerns about increased assimilation and mainstreaming of LGBTQ+ identities [12, 50, 62], stigmatizing unmarried relationships [62], and weakening of unique and valued strengths of LGBTQ+ culture [12]. For example, Bernstein, Harvey, and Naples [96] interviewed 52 Australian LGBTQ+ activists and legislators who worked alongside activists for equal marriage rights. These authors described the “assimilationist dilemma” faced by activists: a concern that gaining acceptance into the mainstream societal institution of marriage would lessen the salience of LGBTQ+ identity and ultimately diminish the richness and strength of LGBTQ+ communities. Another downside of the focus on marriage as a social movement goal was the concern about reinforcing negative heteronormative aspects of marriage rather than challenging them [95].

Four studies explicitly examined possible community level impacts of same-sex marriage. In a mixed-methods study with 115 LGBTQ+ individuals in Massachusetts, participants reported believing that increased acceptance and social inclusion as a result of equal marriage rights might lessen reliance on LGBTQ+-specific activism, events, activities, and venues for social support [13]. However, a majority of study participants (60%) reported participating in LGBTQ+-specific events, activities, or venues “regularly.” A few studies found evidence of concerns that the right to marry could result in marriage being more valued than other relationship configurations [12, 62, 79].

*Local community contexts and extended social networks*. Studies examining the impact of same-sex marriage on sexual minority individuals’ interactions with their extended social networks and in local community contexts yielded mixed results. In an interview study with 19 same-sex couples living in the Netherlands, Badgett [66] found that LGBTQ+ people experienced both direct and indirect increases in social inclusion in their communities and extended social networks as a result of equal marriage rights. For example, direct increases in social inclusion included people making supportive comments to the couple and attending their marriage ceremonies; examples of indirect increases included same-sex spouses being incorporated into family networks [66]. Other studies found mixed or no change in support for LGBTQ+ people and their relationships. Kennedy, Dalla, and Dreesman [61] collected survey data from 210 married LGBTQ+ individuals in midwestern U.S. states, half of whom were living in states with equal marriage rights at the time of data collection. Most participants did not perceive any change in support from their community/social network following legalization of same-sex marriage; other participants reported an increase or mixed support from friends and co-workers. Similarly, Wootton and colleagues interviewed 20 SMW from 15 U.S. states and found positive, neutral, and negative impacts of same-sex marriage on their interactions in work and community contexts [50]. Participants perceived increased positivity about LGBTQ+ issues and more accepting attitudes within their extended social networks and local communities, but also reported hearing negative comments about sexual minority people more frequently and experiencing continued sexual orientation-based discrimination and stigma [50]. Many SMW reported feeling safer and having more positive conversations after Obergefell, but also continued to have concerns about being out at work as a sexual minority person [50].

Two studies examined the experiences of LGBTQ+ people in U.S. states in which same-sex marriage restrictions were decided by voters through ballot measures. These studies documented mixed impacts on participants’ interactions with extended social networks and community. Maisel and Fingerhut [28] surveyed 354 sexual minority adults in California immediately before the vote to restrict recognition of marriage to one man and one woman in the state (Proposition 8) and found that about one-third experienced interactions with social network members that were positive, whereas just under one-third were negative, and the rest were either mixed or neutral. Overall, sexual minority people reported more support than conflict with extended social network members and heterosexual community members over the ballot measure, with friends providing the most support [28]. Social support and solidarity from extended social network members in the face of ballot measures to restrict marriage recognition were also reported in an interview study of 57 same-sex couples residing in one of seven U.S. states that had passed marriage restriction amendments in 2006 [97]. However, some LGBTQ+ people also experienced condemnation and avoidance in their extended social networks [97].

#### Societal level impacts

Sixteen studies examined ways that same-sex marriage influenced societal attitudes about sexual minority individuals or contributed to additional shifts in policies protecting the rights of sexual minority individuals. Findings suggested that the right of same-sex couples to marry had a positive influence on the political and socio-cultural context of sexual minorities’ lives. For example, changes in laws may influence social attitudes or result in LGBTQ positive policy diffusion across states (jurisdictions). There is debate over whether legal changes, such as equal marriage rights, create or are simply reflective of changes in social attitudes toward a group or a social issue [98]. Flores and Barclay [98] theorize four different socio-political responses to changes in marriage laws: backlash, legitimacy, polarization, and consensus. Some scholars argue that changes in law are unlikely to impact social attitudes (consensus), while others argue that legal changes influence the political and social environment that shapes social attitudes. Possible effects range from decreased support for sexual minorities and attempts to rescind rights (backlash) to greater support for the rights of sexual minorities and possible future expansion of rights and protections (legitimacy).

Findings from research generally suggest a positive relationship between same-sex marriage and public support for the overall rights of sexual minorities (legitimacy), and mixed results related to changes in mass attitudes (consensus) [98–106]. For example, in a panel study in Iowa before and after a state Supreme Court ruling in favor of equal marriage rights, Kreitzer and colleagues found that the change in law modified registered voters’ views of the legitimacy of same-sex marriage and that some respondents felt “pressure” to modify or increase their expressed support [102]. Similarly, Flores and Barclay [98] found that people in a state with equal marriage rights showed a greater reduction in anti-gay attitudes than people in a state without equal marriage rights. Studies based on data from European countries also found that more positive attitudes toward sexual minorities were associated with equal marriage rights; improvements in attitudes were not evident in countries without equal marriage rights [9, 105, 106].

There is some evidence to support the third possible socio-political response to changes in marriage laws in Flores and Barclay’s model: increased polarization of the general public’s attitudes toward sexual minorities. Perrin, Smith, and colleagues [107], using successive-independent samples study of conservatives, moderates, and progressives across the U.S. found no overall changes in opinions attitudes about sexual minorities immediately after the Supreme Court decision extending equal marriage rights to all same-sex couples in the U.S. However, analyses by subgroup found that those who were conservative expressed more prejudice toward gay men and lesbians, less support for same-sex marriage, and less support for LGB civil rights immediately after the decision. Similarly, drawing on data from approximately one million respondents in the U.S. who completed implicit and explicit measures of bias against gay men and lesbian women (Project Implicit), Ofosu and colleagues [100] found that implicit bias decreased sharply following Obergefell. However, changes in attitudes were moderated by state laws; respondents in states that already had equal marriage rights for same-sex couples demonstrated decreased bias whereas respondents in states that did not yet have equal marriage rights evidenced increased bias [100]. Using data from the World Values Survey (1989–2014) in European countries, Redman [103] found that equal marriage rights were associated with increases in positive opinions about sexual minorities, but that the increase was driven largely by those who already held positive views.

Little support has been found for the hypothesis that the extension of equal marriage rights would be followed by a backlash of sharp negative shifts in mass attitudes and public policy [98, 108, 109]. For example, a general population survey in one relatively conservative U.S. state (Nebraska) found public support for same-sex marriage was higher after the Supreme Court ruling than before, suggesting no backlash in public opinion [108]. Similarly, Bishin and colleagues [109], using both an online survey experiment and analysis of data from a U.S. public opinion poll (National Annenberg Election Studies) before and after three relevant policy events, found little change in public opinion in response to simulated or actual policy changes.

Although equal marriage rights confer parental recognition rights, there are still legal challenges and disparate rulings and interpretations about some family law issues [77, 110, 111]. For example, some states in the U.S. have treated the parental rights of same-sex couples differently than those of different-sex (presumed heterosexual) couples. Both members of a same-sex couple have traditionally not been automatically recognized as parents of a child born or adopted within the relationship. However, the presumptions of parenthood after same-sex marriage was legalized have forced states to treat both members of same-sex couples as parents irrespective of method of conception or adoption status [112]. Still, results from a cross-national study of laws, policies, and legal recognition of same-sex relationships suggests that parental rights are recognized in some jurisdictions but not others [111].

### Aim 2: SMW-specific findings and differences by gender

A total of 13 studies included in this review conducted SMW-specific analyses or compared SMW and SMM’s perceptions and experiences of same-sex marriage and equal marriage rights. In studies that included only SMW [50, 64, 68, 77, 81, 86, 89, 91], findings emphasized the importance of relational and interpersonal impacts of same-sex marriage. Examples include creating safety for sexual identity disclosure and visibility [68, 81], providing legal protections in relation to partners and/or children [77, 81], offering social validation [86, 89], and reducing stigma in larger community contexts [50, 64]. Relational themes centered on concerns and distress when experiencing rejection or absence of support from family members or extended social networks [50, 81, 86, 89, 91].

Two of the studies of SMW documented sexual identity and gender identity differences in interpersonal experiences associated with same-sex marriage [86, 89]. Lannutti’s interview study of the experiences of 26 married or engaged SMW couples with different sexual identities (bisexual-lesbian couples) revealed how the right to marry made them feel more connected to LGBTQ+ communities through activism and being “counted” as a same-sex married couple. However, same-sex marriage made some bisexual women feel more invisible within LGBTQ+ communities [89]. Scott and Theron [86] found that married lesbian women and cisgender women partners of transmasculine individuals (i.e., masculine-identifying transgender individuals) faced different challenges as they navigated through gendered social expectations and made choices about conforming or rejecting heteronormativity.

Only five of the studies focusing on psychosocial impacts of equal marriage rights explicitly examined potential differences by sex [28, 66, 73, 76, 95]. Some studies found perceptions of greater social inclusion [66], or feelings of ambivalence (simultaneously holding positive, negative, and critical perspectives about marriage as an institution) [95] that were similar among SMW and SMM. Maisel and Fingerhut’s study of consequences of a state-level campaign to restrict marriage rights [28] showed that SMW and SMM experienced similar negative impacts on personal well-being and interactions with extended social networks. However, Lee found that, compared with Black SMM, Black SMW perceived same-sex marriage to have a larger impact on their lives [73]. Other studies found that SMW were more likely than SMM to report positive perceptions of same-sex marriage, possibly because they are more likely than SMM to have children and to be concerned about parental protections [73, 95]. SMW and SMM may be differentially impacted by interpersonal stigma despite equal marriage rights. For example, one study found that SMW experienced higher levels of distress than SMM when their relationships were not treated as equal to heterosexuals’ [76].

## Discussion

Overall, findings from this scoping review suggest that psychosocial impacts of equal marriage rights among sexual minorities are apparent at all levels of our social-ecological and stigma framework. Sexual minority-specific stigma occurs on multiple levels (e.g., individual, interpersonal, and structural simultaneously and changes in social policies have cascading effects on sexual minority individuals’ experiences at each level. Generally, equal marriage rights had a positive impact on perceptions of social acceptance and social inclusion for sexual minority individuals, couples, and the LGBTQ+ community as a whole. However, many studies described mixed, ambivalent, or complicated perceptions of same-sex marriage, as well as stigmatizing interactions that were unaffected or exacerbated by equal marriage rights.

Although research does not unequivocally suggest the presence of a backlash in public opinion after equal marriage rights, there has been an increase in laws and policies at the U.S. state and federal levels that explicitly allow for religious-belief-based denial of services to sexual minority individuals and same-sex couples. For example, by 2017, 12 states in the U.S. enacted laws permitting the denial of services (e.g., allowing government officials to refuse to issue same-sex marriage licenses, allowing magistrates to refuse to perform same-sex marriages, and permitting adoption and child welfare agencies to refuse same-sex couples’ adoption or fostering children) based on religious beliefs [113]. Research has documented negative health and psychological outcomes among sexual minorities living in U.S. states with policies that permit denial of services to sexual or gender minorities [114, 115] and in states that do not have legal protections against discrimination [38, 116, 117]. Additional research is needed to examine how changes in local or national laws impact the health and well-being of sexual and gender minorities—particularly over the long term.

### Gaps & future research needs

Research is limited in terms of examining how same-sex marriage may differentially impact sexual minority individuals based on sex, gender identity, or race/ethnicity. Only 14 studies included in this review addressed the psychosocial impacts of same-sex marriage among SMW. More research is needed to understand the unique experiences and psychosocial impact of same-sex marriage for SMW and SMM. Further, many study samples were largely homogenous and included an overwhelming majority of White participants. The few studies with substantial sample sizes of people of color, and that compared people of color to White people, found differences by race in perceived impact of same-sex marriage [64, 67, 73], demonstrating the need for additional work in this area.

There were also very few studies in this review that explored differences by sexual identity (e.g., monosexual vs. plurisexual), gender identity (e.g., transgender vs. cisgender), gender expression (e.g., masculine vs. feminine presentation), or differences based on sex/gender of participants’ partners. Although transgender and nonbinary individuals were included in eight studies, five provided only descriptive information and only three described any unique findings from transgender study participants. For example, McGuffey [72] found that transgender individuals who identified as heterosexual described same-sex marriage rights as less relevant than issues of gender identity and expression and Hull found that cisgender sexual minority men generally expressed more enthusiasm about marriage than both cisgender women and transgender individuals [69]. Transgender and nonbinary individuals who perceive positive impacts of equal marriage rights may still experience challenges in navigating heteronormative and cisnormative expectations [72, 86]. Other qualitative studies documented concerns that LGBTQ+ advocacy efforts, once marriage rights were secured, might fail to address rights and protections for transgender and nonbinary individuals [62, 69]. Future studies that include the voices of transgender and nonbinary individuals are needed to better understand perceptions across both sexual and gender identities [118].

There is limited research on immediate and extended family members’ perceptions of equal marriage rights. There is also a need for prospective studies that examine whether familial acceptance increases over time. Many studies did not account for differences in LGBTQ+ identity salience and connection to LGBTQ+ and other communities, which may influence differences in perceptions and reactions to same-sex marriage.

The majority of studies (43 of 59) we reviewed were conducted in the U.S. Eleven of these collected data after Obergefell (June 25, 2015). Only two used longitudinal research designs that included data collection before and after national same-sex marriage legalization [44, 107]. The legal and social landscapes have changed since this time and there is a need for re-assessment of the impact of same-sex marriage over multiple future timepoints.

### Limitations

Although this scoping review used a systematic approach and, to our knowledge, is novel in its focus on impact of equal marriage rights on sexual minorities’ personal lives, interpersonal relationships, and social/community contexts, we acknowledge several limitations. We did not conduct a search of grey literature (e.g., reports, policy literature, working papers) or books and, consequently, likely excluded some scholarly work aligned with our focus. Our inclusion criteria of only peer-reviewed studies may have led us to exclude dissertations that focus on emerging areas of research, such as differences by gender identity, sexual identity, or race and ethnicity. As with all scoping reviews, studies may have been missed because of the search strategy. For example, it is possible that relevant studies were indexed in databases not used in our review. We also restricted our review to English language literature, excluding potentially relevant studies published in other languages. Studies in other languages may provide useful insights from other countries where English is not widely used. Although we focused exclusively on empirical studies, we did not assess the quality of the studies. Findings of the review are also limited by the collective body of research questions, designs, and analyses that have been pursued. For example, as noted above, few studies explored psychosocial impacts of same-sex marriage among SMW or explored differences by sex; consequently we were limited in our ability to address our second research aim.

## Conclusion

This scoping review identified and described psychosocial impacts of equal marriage rights among sexual minority adults and explored potential SMW-specific experiences and differences by sex. Our results highlight four points. First, equal marriage rights are associated with a wide range of positive impacts on the psychological and social well-being of sexual minority adults. Second, the potential positive impacts of equal marriage rights are amplified or weakened by the presence or absence of stigma in interpersonal interactions and in the larger political and social environment. Third, although there is a growing body of global research on the impact of same-sex marriage, most studies have been conducted in the U.S. Cross-cultural studies can improve understanding of individual, interpersonal, and community level impacts of same-sex marriage in different cultural contexts. Fourth, given indications of differences between SMW and SMM in perceived impact of same-sex marriage, there is a need for research that examines the specific perspectives of SMW and that explores possible differences in perspectives and experiences by sex. Research is also needed to understand differences based on race/ethnicity, gender identity, and age. The right of same-sex couples to marry does not merely address the concerns of sexual minorities, it aims to right a far bigger wrong: the exclusion of some individuals from one of the most important institutions in social life.

## Supporting information

S1 Table Articles included in scoping review on the psychosocial impact of equal marriage rights among sexual minority adults. (PDF)

S1 Checklist Preferred Reporting Items for Systematic reviews and Meta-Analyses extension for scoping reviews (PRISMA-ScR) checklist. (PDF)

S1 Text Definitions. (DOCX)

## References

[1] Pew Research Center. Same-sex marriage around the world. Fact Sheet [Internet]. 2019; (10 28). Available from: https://www.pewforum.org/fact-sheet/gay-marriage-around-the-world/.

[2] Hatzenbuehler ML, Link B. Introduction to the special issues on structural stigma and health. Social Science and Medicine. 2014;103:1–6. 10.1016/j.socscimed.2013.12.017 24445152

[3] Herek GM. Sexual stigma and sexual prejudice in the United States: A conceptual framework. In: Hope DA, Editor. Contemporary perspectives on lesbian, gay, and bisexual identities. New York: Springer; 2009. p. 65–111. 10.1007/978-0-387-09556-1_4 19230525

[4] Hatzenbuehler ML. Structural stigma and the health of lesbian, gay, and bisexual populations. Current Directions in Psychological Science. 2014;23(2):127–32.

[5] Hatzenbuehler ML, McLaughlin KA, Keyes KM, Hasin DS. The impact of institutional discrimination on psychiatric disorders in lesbian, gay, and bisexual populations: A prospective study. American Journal of Public Health. 2010;100(3):452–9. 10.2105/AJPH.2009.168815 20075314 PMC2820062

[6] Hatzenbuehler ML, Phelan JC, Link BG. Stigma as a fundamental cause of population health inequalities. American Journal of Public Health. 2013;103(5):813–21. 10.2105/AJPH.2012.301069 23488505 PMC3682466

[7] Hatzenbuehler ML. Structural stigma: Research evidence and implications for psychological science. American Psychologist. 2016;71(8):742–51. 10.1037/amp0000068 27977256 PMC5172391

[8] Hatzenbuehler ML, Bränström R, Pachankis JE. Societal-level explanations for reductions in sexual orientation mental health disparities: Results from a ten-year, population-based study in Sweden. Stigma and Health. 2018;3(1):16–26.

[9] Herdt G, Kertzner R. I do, but I can’t: The impact of marriage denial on the mental health and sexual citizenship of lesbians and gay men in the United States. Sexuality Research and Social Policy. 2006;3(1):33–49.

[10] Lannutti PJ. Experiencing same-sex marriage: Individual, couples, and social networks. New York: Peter Lang; 2014.

[11] Drabble LA, Wootton AR, Veldhuis CB, Perry E, Riggle ED, Trocki KF, et al. It’s complicated: The impact of marriage legalization among sexual minority women and gender diverse individuals in the United States. Psychology of Sexual Orientation and Gender Diversity. 2020;7(4):396–406. 10.1037/sgd0000375 33778093 PMC7992926

[12] Lannutti PJ. Security, recognition, and misgivings: Exploring older same-sex couples’ experiences of legally recognized same-sex marriage. Journal of Social and Personal Relationships. 2011;28(1):64–82.

[13] Ocobock A. Status or access? The impact of marriage on lesbian, gay, bisexual, and queer community change. Journal of Marriage and Family. 2018;80(2):367–82.

[14] Kuyper L, de Roos S, Iedema J, Stevens G. Growing up with the right to marry: Sexual attraction, substance use, and well-being of Dutch adolescents. Journal of Adolescent Health. 2016;59(3):276–82. 10.1016/j.jadohealth.2016.05.010 27423901

[15] Buffie WC. Public health implications of same-sex marriage. American Journal of Public Health. 2011;101(6):986–90. 10.2105/AJPH.2010.300112 21493934 PMC3093259

[16] Gonzales G. Same-sex marriage—A prescription for better health. New England Journal of Medicine. 2014;370(15):1373–6. 10.1056/NEJMp1400254 24716677

[17] Kealy-Bateman W, Pryor L. Marriage equality is a mental health issue. Australasian Psychiatry. 2015;23(5):540–3. 10.1177/1039856215592318 26139705

[18] Kertzner RM. A mental health research perspective on marital rights and civil marriage for lesbians and gay men. Journal of Gay & Lesbian Mental Health. 2012;16(2):136–45.

[19] Perone AK. Health implications of the supreme court’s Obergefell vs Hodges marriage equality decision. LGBT Health. 2015;2(3):196–9. 10.1089/lgbt.2015.0083 26788668 PMC4713052

[20] Tuller D. The health effects of legalizing same-sex marriage. Health Affairs. 2017;36(6):978–81. 10.1377/hlthaff.2017.0502 28583954

[21] Kail BL, Acosta KL, Wright ER. State-level marriage equality and the health of same-sex couples. American Journal of Public Health. 2015;105(6):1101–5. 10.2105/AJPH.2015.302589 25880959 PMC4431106

[22] Raifman J, Moscoe E, Austin B, McConnell M. Difference-in-differences analysis of the association between state same-sex marriage policies and adolescent suicide attempts. JAMA Pediatrics. 2017;171(4):350–6. 10.1001/jamapediatrics.2016.4529 28241285 PMC5848493

[23] Kennedy HR, Dalla RL. “It may be legal, but it is not treated equally”: Marriage equality and well-being implications for same-sex couples. Journal of Gay and Lesbian Social Services. 2020;32(1):67–98.

[24] Hatzenbuehler ML, O’Cleirigh C, Grasso C, Mayer K, Safren S, Bradford J. Effect of same-sex marriage laws on health care use and expenditures in sexual minority men: A quasi-natural experiment. American Journal of Public Health. 2012;102(2):285–91. 10.2105/AJPH.2011.300382 22390442 PMC3484969

[25] Fingerhut AW, Riggle EDB, Rostosky SS. Same‐sex marriage: The social and psychological implications of policy and debates. Journal of Social Issues. 2011;67(2):225–41.

[26] Flores AR, Hatzenbuehler ML, Gates GJ. Identifying psychological responses of stigmatized groups to referendums. PNAS Proceedings of the National Academy of Sciences of the United States of America. 2018;115(15):3816–21. 10.1073/pnas.1712897115 29581304 PMC5899433

[27] Frost DM, Fingerhut AW. Daily exposure to negative campaign messages decreases same-sex couples’ psychological and relational well-being. Group Processes & Intergroup Relations. 2016;19(4):477–92.

[28] Maisel NC, Fingerhut AW. California’s ban on same‐sex marriage: The campaign and its effects on gay, lesbian, and bisexual individuals. Journal of Social Issues. 2011;67(2):242–63.

[29] Riggle ED, Rostosky SS, Horne SG. Marriage amendments and lesbian, gay, and bisexual individuals in the 2006 election. Sexuality Research & Social Policy. 2009;6(1):80–9.

[30] Rostosky SS, Riggle EDB, Horne SG, Denton FN, Huellemeier JD. Lesbian, gay, and bisexual individuals’ psychological reactions to amendments denying access to civil marriage. American Journal of Orthopsychiatry. 2010;80(3):302–10. 10.1111/j.1939-0025.2010.01033.x 20636935

[31] Tatum AK. The interaction of same-sex marriage access with sexual minority identity on mental health and subjective wellbeing. Journal of Homosexuality. 2017;64(5):638–53. 10.1080/00918369.2016.1196991 27269121

[32] Perales F, Todd A. Structural stigma and the health and wellbeing of Australian LGB populations: Exploiting geographic variation in the results of the 2017 same-sex marriage plebiscite. Social Science & Medicine. 2018;208:190–9. 10.1016/j.socscimed.2018.05.015 29853260

[33] Coulter RW, Kenst KS, Bowen DJ. Research funded by the National Institutes of Health on the health of Lesbian, Gay, Bisexual, and Transgender populations. American Journal of Public Health. 2014;104(2):e105–e12. 10.2105/AJPH.2013.301501 24328665 PMC3935708

[34] Voyles CH, Sell RL. Continued disparities in lesbian, gay, and bisexual research funding at NIH. American Journal of Public Health. 2015;105(S3):e1–e2. 10.2105/AJPH.2014.302265 PMC4455511 25905824

[35] Umberson D, Kroeger RA. Gender, marriage, and health for same-sex and different-sex couples: The future keeps arriving. In: McHale SM, King V, Van Hook J, Booth A, editors. Gender and Couple Relationships. National Symposium on Family Issues, Vol 6. Switzerland: Springer International; 2016. p. 189–213.

[36] Homan P. Structural sexism and health in the United States: A new perspective on health inequality and the gender system. American Sociological Review. 2019;84(3):486–516.

[37] Carpenter C, Eppink ST, Gonzales Jr G, McKay T. Effects of Access to Legal Same-Sex Marriage on Marriage and Health: Evidence from BRFSS. National Bureau of Economic Research; 2018. Contract No.: No. w24651.

[38] Gonzales G, Ehrenfeld JM. The association between state policy environments and self-rated health disparities for sexual minorities in the United States. International Journal of Environmental Research and Public Health. 2018;15(6):Article number 1136. 10.3390/ijerph15061136 29857580 PMC6024973

[39] Elwood WN, Irvin VL, Sun Q, Breen N. Measuring the influence of legally recognized partnerships on the health and well-being of same-sex couples: Utility of the California Health Interview Survey. LGBT Health. 2017;4(2):153–60. 10.1089/lgbt.2015.0085 28207297 PMC5404247

[40] Hsieh N. Mental health disparities by sexual orientation in the US: Current patterns and recent trends. EurAmerica. 2019;49(2):201–41.

[41] Goldsen J, Bryan AEB, Kim H-J, Muraco A, Jen S, Fredriksen-Goldsen KI. Who says I do: The changing context of marriage and health and quality of life for LGBT older adults. The Gerontologist. 2017;57(Suppl 1):S50–S62. 10.1093/geront/gnw174 28087795 PMC5241756

[42] Hatzenbuehler ML, Pachankis JE. Stigma and minority stress as social determinants of health among lesbian, gay, bisexual, and transgender youth: Research evidence and clinical implications. Pediatric Clinics of North America. 2016;63(6):985–97. 10.1016/j.pcl.2016.07.003 27865340

[43] Rostosky SS, Riggle ED. Same-sex relationships and minority stress. Current Opinion in Psychology. 2016;13:29–38. 10.1016/j.copsyc.2016.04.011 28813290

[44] Ogolsky BG, Monk JK, Rice TM, Oswald RF. Personal well-being across the transition to marriage equality: A longitudinal analysis. Journal of Family Psychology. 2019;33(4):422–32. 10.1037/fam0000504 30730185

[45] Richman LS, Hatzenbuehler ML. A multilevel analysis of stigma and health: Implications for research and policy. Policy Insights from the Behavioral and Brain Sciences. 2014;1(1):213–21.

[46] Fredriksen-Goldsen KI, Simoni JM, Kim H-J, Lehavot K, Walters KL, Yang J, et al. The health equity promotion model: Reconceptualization of lesbian, gay, bisexual, and transgender (LGBT) health disparities. American Journal of Orthopsychiatry. 2014;84(6):653–63. 10.1037/ort0000030 PMC4350932 25545433

[47] Riggle EDB, Wickham RE, Rostosky SS, Rothblum ED, Balsam KF. Impact of civil marriage recognition for long-term same-sex couples. Sexuality Research & Social Policy: A Journal of the NSRC. 2017;14(2):223–32.

[48] Williams SL, Mann AK. Sexual and gender minority health disparities as a social issue: How stigma and intergroup relations can explain and reduce health disparities. Journal of Social Issues. 2017;73(3):450–61.

[49] Williams SL, Mann AK, Fredrick EG. Proximal minority stress, psychosocial resources, and health in sexual minorities. Journal of Social Issues. 2017;73(3):529–44.

[50] Wootton AR, Drabble LA, Riggle EDB, Veldhuis CB, Bitcon C, Trocki KF, et al. Impacts of marriage legalization on the experiences of sexual minority women in work and community contexts. Journal of GLBT Family Studies. 2019;15(3):211–34 10.1080/1550428X.2018.1474829 31080374 PMC6508647

[51] Hatzenbuehler ML, Flores AR, Gates GJ. Social attitudes regarding same‐sex marriage and LGBT health disparities: Results from a national probability sample. Journal of Social Issues. 2017;73(3):508–28.

[52] Woodford MR, Paceley MS, Kulick A, Hong JS. The LGBQ cocial climate matters: Policies, protests, and placards and psychological well-being among LGBQ emerging adults. Journal of Gay & Lesbian Social Services. 2015;27(1):116–41.

[53] Monk JK, Ogolsky BG. Contextual relational uncertainty model: Understanding ambiguity in a changing sociopolitical context of marriage. Journal of Family Theory & Review. 2019;11(2):243–61.

[54] Veldhuis CB, Drabble L, Riggle EDB, Wootton AR, Hughes TL. “We won’t go back into the closet now without one hell of a fight”: Effects of the 2016 presidential election on sexual minority women’s and gender minorities’ stigma-related concerns. Sexuality Research and Social Policy. 2018;15(1):12–24.

[55] Pham MT, Rajić A, Greig JD, Sargeant JM, Papadopoulos A, McEwen SA. A scoping review of scoping reviews: Advancing the approach and enhancing the consistency. Research synthesis methods. 2014;5(4):371–85. 10.1002/jrsm.1123 26052958 PMC4491356

[56] Arksey H, O’Malley L. Scoping studies: Towards a methodological framework. International Journal of Social Research Methodology. 2005;8(1):19–32.

[57] Munn Z, Peters MD, Stern C, Tufanaru C, McArthur A, Aromataris E. Systematic review or scoping review? Guidance for authors when choosing between a systematic or scoping review approach. BMC Medical Research Methodology [Internet]. 2018; 18(1): Article 143. 10.1186/s12874-018-0611-x PMC6245623 30453902

[58] Tricco AC, Lillie E, Zarin W, O’Brien KK, Colquhoun H, Levac D, et al. PRISMA extension for scoping reviews (PRISMA-ScR): checklist and explanation. Annals of internal medicine. 2018;169(7):467–73. 10.7326/M18-0850 30178033

[59] Haas SM, Whitton SW. The significance of living together and importance of marriage in same-sex couples. Journal of Homosexuality. 2015;62(9):1241–63. 10.1080/00918369.2015.1037137 25848857

[60] Jowett A, Peel E. ‘A question of equality and choice’: Same-sex couples’ attitudes towards civil partnership after the introduction of same-sex marriage. Psychology & Sexuality. 2017;8(1–2):69–80.

[61] Kennedy HR, Dalla RL, Dreesman S. "We are two of the lucky Ones": Experiences with marriage and wellbeing for aame-sex couples. Journal of Homosexuality. 2018;65(9):1207–31. 10.1080/00918369.2017.1407612 29199908

[62] Lannutti PJ. For better or worse: Exploring the meanings of same-sex marriage within the lesbian, gay, bisexual and transgendered community. Journal of Social and Personal Relationships. 2005;22(1):5–18.

[63] Lannutti PJ. The influence of same-sex marriage on the understanding of same-sex relationships. Journal of Homosexuality. 2007;53(3):135–51. 10.1300/J082v53n03_08 18032290

[64] Everett BG, Hatzenbuehler ML, Hughes TL. The impact of civil union legislation on minority stress, depression, and hazardous drinking in a diverse sample of sexual-minority women: A quasi-natural experiment. Social Science & Medicine. 2016;169:180–90. 27733300 10.1016/j.socscimed.2016.09.036 PMC5364018

[65] Balsam KF, Rothblum ED, Wickham RE. Longitudinal predictors of relationship dissolution among same-sex and heterosexual couples. Couple and Family Psychology: Research and Practice. 2017;6(4):247–57.

[66] Badgett MVL. Social inclusion and the value of marriage equality in Massachusetts and the Netherlands. Journal of Social Issues. 2011;67(2):316–34.

[67] Metheny N, Stephenson R. Political environment and perceptions of social inclusion after nationwide marriage equality among partnered men who have sex with men in the USA. Sexuality Research & Social Policy. 2019;16(4):521–8. 10.1007/s13178-018-0357-6 31798756 PMC6889050

[68] Charlton BM, Corliss HL, Spiegelman D, Williams K, Austin SB. Changes in reported sexual orientation following US states recognition of same-sex couples. American Journal of Public Health. 2016;106(12):2202–4. 10.2105/AJPH.2016.303449 27736213 PMC5104997

[69] Hull KE. Same-sex marriage: Principle versus practice. International Journal of Law Policy and the Family. 2019;33(1):51–74.

[70] Reynolds R, Robinson S. Marriage as a marker of secular inclusion? Oral history and lesbian and gay narratives on marriage in contemporary Australia. Journal of Religious History. 2019;43(2):269–84.

[71] Philpot SP, Ellard J, Duncan D, Dowsett GW, Bavinton BR, Down I, et al. Gay and bisexual men’s interest in marriage: An Australian perspective. Culture, Health & Sexuality. 2016;18(12):1347–62. 10.1080/13691058.2016.1184314 27240739

[72] McGuffey CS. Intersectionality, cognition, disclosure and Black LGBT views on civil rights and marriage equality: Is gay the new Black? Du Bois Review: Social Science Research on Race. 2018;15(2):441–65.

[73] Lee J. Black LGB identities and perceptions of same-sex marriage. Journal of Homosexuality. 2018;65(14):2005–27. 10.1080/00918369.2017.1423214 29319435

[74] Rostosky SS, Riggle EDB, Rothblum ED, Balsam KF. Same-sex couples’ decisions and experiences of marriage in the context of minority stress: Interviews from a population-based longitudinal study. Journal of Homosexuality. 2016;63(8):1019–40. 10.1080/00918369.2016.1191232 27191207

[75] Shulman JL, Gotta G, Green R-J. Will marriage matter? Effects of marriage anticipated by same-sex couples. Journal of Family Issues. 2012;33(2):158–81.

[76] LeBlanc AJ, Frost DM, Bowen K. Legal marriage, unequal recognition, and mental health among same-sex couples. Journal of Marriage and Family. 2018;80(2):397–408. 10.1111/jomf.12460 29755137 PMC5942902

[77] DiGregorio N. Same-sex marriage policies and lesbian family life. Sexuality Research & Social Policy. 2016;13(1):58–72.

[78] Shulman JL, Weck V, Schwing S, Smith T, Coale E. The push-pull of policy pressure: A qualitative exploration of the experiences of same-sex marriage policies among non-metropolitan GLB individuals. Journal of GLBT Family Studies. 2009;5(4):340–65.

[79] Lannutti PJ. Committed, unmarried same-sex couples and their social networks in the United States: Relationships and discursive strategies. Journal of Homosexuality. 2018;65(9):1232–48. 10.1080/00918369.2017.1411690 29185872

[80] Clark JB, Riggle EDB, Rostosky SS, Rothblum ED, Balsam KF. Windsor and Perry: Reactions of siblings in same-sex and heterosexual couples. Journal of Homosexuality. 2015;62(8):993–1008. 10.1080/00918369.2015.1039360 25865954

[81] Balsam KF, Rostosky SS, Riggle EDB. Breaking up is hard to do: Women’s experience of dissolving their same-sex relationship. Journal of Lesbian Studies. 2017;21(1):30–46. 10.1080/10894160.2016.1165561 27602487

[82] Alderson KG. A phenomenological investigation of same-sex marriage. Canadian Journal of Human Sexuality. 2004;13(2):107–22.

[83] MacIntosh H, Reissing ED, Andruff H. Same-sex marriage in Canada: The impact of legal marriage on the first cohort of gay and lesbian Canadians to wed. Canadian Journal of Human Sexuality. 2010;19(3):79–90.

[84] Goodwin C, Butler C. Legitimate love: The meaning of civil partnership for the positioning of lesbian and gay people in society. Sexual and Relationship Therapy. 2009;24(3–4):235–48.

[85] Thomas M. Atrocity stories and triumph stories: Using couple narratives to evaluate same-sex marriage and civil partnership. Narrative Inquiry. 2014;24(2):200–17.

[86] Scott J, Theron L. The promise of heteronormativity: Marriage as a strategy for respectability in South Africa. Sexualities. 2019;22(3):436–51.

[87] Lannutti PJ. Same-sex marriage and privacy management: Examining couples’ communication with family members. Journal of Family Communication. 2013;13(1):60–75.

[88] Schecter E, Tracy AJ, Page KV, Luong G. Shall we marry? Legal marriage as a commitment event in same-sex relationships. Journal of Homosexuality. 2008;54(4):400–22. 10.1080/00918360801991422 18826168

[89] Lannutti PJ. ’This is not a lesbian wedding’: Examining same-sex marriage and bisexual-lesbian couples. Journal of Bisexuality. 2007;7(3–4):237–60.

[90] Ocobock A. The power and limits of marriage: married gay men’s family relationships. Journal of Marriage and Family. 2013;75(1):191–205.

[91] Riggle EDB, Drabble L, Veldhuis C, Wootton A, Hughes TL. The impact of marriage equality on sexual minority women’s relationships with their families of origin. Journal of Homosexuality. 2018;65(9):1190–206. 10.1080/00918369.2017.1407611 29161223

[92] Lannutti PJ. Attractions and obstacles while considering legally recognized same-sex marriage. Journal of GLBT Family Studies. 2008;4(2):245–64.

[93] Lannutti PJ. GLBTQ people who decided to marry after the 2016 US election: Reasons for and meanings of marriage. Journal of GLBT Family Studies. 2018;14(1–2):85–100.

[94] Vuckovic Juros T. Transformative power of same-sex marriage and non-heterosexual reproductivity How parents of glb offspring adjust to their marriage and children. Journal of GLBT Family Studies. 2019.

[95] Bosley-Smith ER, Reczek C. Before and after ’I Do’: Marriage processes for mid-life gay and lesbian married couples. Journal of Homosexuality. 2018;65(14):1985–2004. 10.1080/00918369.2017.1423213 29611778 PMC6219458

[96] Bernstein M, Harvey B, Naples NA. Marriage, the Final Frontier? Same-Sex Marriage and the Future of the Lesbian and Gay Movement. Sociological Forum. 2018;33(1):30–52.

[97] Lannutti PJ. Examining communication about marriage amendments: Same‐sex couples and their extended social networks. Journal of Social Issues. 2011;67(2):264–81.

[98] Flores AR, Barclay S. Backlash, consensus, legitimacy, or polarization: The effect of same-sex marriage policy on mass attitudes. Political Research Quarterly. 2016;69(1):43–56.

[99] Hooghe M, Meeusen C. Is same-sex marriage legislation related to attitudes toward homosexuality?: Trends in tolerance of homosexuality in European countries between 2002 and 2010. Sexuality Research & Social Policy. 2013;10(4):258–68.

[100] Ofosu EK, Chambers MK, Chen JM, Hehman E. Same-sex marriage legalization associated with reduced implicit and explicit antigay bias. PNAS Proceedings of the National Academy of Sciences of the United States of America. 2019;116(18):8846–51. 10.1073/pnas.1806000116 30988191 PMC6500130

[101] Sansone D. Pink work: Same-sex marriage, employment and discrimination. Journal of Public Economics. 2019;180:20.

[102] Kreitzer RJ, Hamilton AJ, Tolbert CJ. Does policy adoption change opinions on minority rights? The effects of legalizing same-sex marriage. Political Research Quarterly. 2014;67(4):795–808.

[103] Redman SM. Effects of same-sex legislation on attitudes toward homosexuality. Political Research Quarterly. 2018;71(3):628–41.

[104] Tankard ME, Paluck EL. The effect of a Supreme Court decision regarding gay marriage on social norms and personal attitudes. Psychological Science. 2017;28(9):1334–44. 10.1177/0956797617709594 28758838

[105] Abou-Chadi T, Finnigan R. Rights for same-sex couples and public attitudes toward gays and lesbians in Europe. Comparative Political Studies. 2019;52(6):868–95.

[106] Aksoy CG, Carpenter CS, De Haas R, Tran KD. Do laws shape attitudes? Evidence from same-sex relationship recognition policies in Europe. European Economic Review. 2020;124:Article 103399.

[107] Perrin PB, Smith ER, Trujillo MA, Rabinovitch A, Coy AE. Differential effects of the US Supreme Court’s same-sex marriage decision on national support for lesbian, gay, and bisexual civil rights and sexual prejudice. Sexuality Research & Social Policy. 2018;15(3):342–52.

[108] Kazyak E, Stange M. Backlash or a positive response?: Public opinion of LGB issues After Obergefell v. Hodges. Journal of Homosexuality. 2018;65(14):2028–52. 10.1080/00918369.2017.1423216 29319437

[109] Bishin BG, Hayes TJ, Incantalupo MB, Smith CA. Opinion backlash and public attitudes: Are political advances in gay rights counterproductive? American Journal of Political Science. 2016;60(3):625–48.

[110] Gash A, Raiskin J. Parenting without protection: How legal status ambiguity affects lesbian and gay parenthood. Law and Social Inquiry-Journal of the American Bar Foundation. 2018;43(1):82–118.

[111] Bernstein M, Naples NA, Harvey B. The meaning of marriage to same-sex families: Formal partnership, parenthood, gender, and the welfare state in international perspective. Social Politics. 2016;23(1):3–39.

[112] Chauveron LM, Alvarez A, van Eeden-Moorefield B. The co-evolution of marriage and parental rights of gays and lesbians. Journal of GLBT Family Studies. 2017;13(2):114–36.

[113] Movement Advancement Project. Equality Maps: Religious Exemption Laws 2020 [Available from: https://www.lgbtmap.org/equality-maps/religious_exemption_laws.

[114] Raifman J, Moscoe E, Austin SB, Hatzenbuehler ML, Galea S. Association of state laws permitting denial of services to same-sex couples with mental distress in sexual minority adults: A difference-in-difference-in-differences analysis. JAMA Psychiatry. 2018;75(7):671–7. 10.1001/jamapsychiatry.2018.0757 29799924 PMC6129969

[115] Blosnich JR, Cassese EC, Friedman MR, Coulter RWS, Sang JM, Matthews DD, et al. Religious freedom restoration acts and sexual minority population health in the United States. American Journal of Orthopsychiatry. 2019;89(6):1–8. 10.1037/ort0000349 30247051 PMC6431586

[116] Solazzo A, Brown TN, Gorman BK. State-level climate, anti-discrimination law, and sexual minority health status: An ecological study. Social Science and Medicine. 2018;196:158–65. 10.1016/j.socscimed.2017.11.033 29190536

[117] Hatzenbuehler ML, McKetta S, Goldberg N, Sheldon A, Friedman SR, Cooper HL, et al. Trends in state policy support for sexual minorities and HIV-related outcomes among men who have sex with men in the United States, 2008–2014. JAIDS Journal of Acquired Immune Deficiency Syndromes. 2020;85(1):39–45. 10.1097/QAI.0000000000002395 32398556 PMC7429252

[118] Shultz JW, Shultz K. Queer and trans after Obergefell v. Hodges: An autoethnographic oral history. Humboldt Journal of Social Relations. 2016;1(38):46–61.

