# Supplementary material for: Perceived psychosocial impacts of legalized same-sex marriage: A scoping review of sexual minority adults’ experiences
Source: PLoS One. 2021 May 6;16(5):e0249125. doi: 10.1371/journal.pone.0249125 (PMC8101749; doi:10.1371/journal.pone.0249125)
Supplement: S1 Table — (PDF) [file pone.0249125.s001.pdf]

S1 Table: Articles included in scoping review on the psychosocial impact of equal marriage rights among sexual minority adults

General Information					Analysis performed		Primary classification in scoping review*				
**Citation**	**Location**	**Methods**	**Participant gender(s)**	**Percent people of color**	**SMW-specific**	**Race/ ethnicity-specific**	**Indivi-dual**	**Couple**	**Family**	**Com-munity**	**Society**
(Abou-Chadi & Finnigan, 2019)	Europe	Quant	Mixed	N/A or not measured							X
(Aksoy, Carpenter, De Haas, & Tran, 2020)	Europe	Quant	N/A or not measured	N/A or not measured							X
(Alderson, 2004)	Canada	Qual	Mixed	Less than 25%				X			
(Badgett, 2011)	Multiple countries	Mixed	Mixed	Less than 25%	X	X	X		X	X	
(Balsam, Rostosky, & Riggle, 2017)	U.S. [2]	Qual	Women only	Less than 25%	X			X			
(Bernstein, Naples, & Harvey, 2016)	Multiple countries	Other	Mixed	N/A or not measured							X
(Bernstein, Harvey, & Naples, 2018)	Australia /NZ	Qual	Mixed	N/A or not measured						X	
(Bishin, Hayes, Incantalupo, & Smith, 2016)	U.S. [1]	Quant	Mixed	Less than 25%							X
(Bosley-Smith & Reczek, 2018)	U.S. [2]	Qual	Mixed	Less than 25%	X					X	
(Charlton, Corliss, Spiegelman, Williams, & Austin, 2016)	U.S. [1]	Quant	Women only	N/A or not measured	X		X				
(Chauveron, Alvarez, & van Eeden-Moorefield, 2017)	U.S. [4]	Other	N/A or not measured	N/A or not measured							X

Citation	Location	Methods	Participant gender(s)	Percent people of color	SMW-specific	Race/ ethnicity-specific	Indivi-dual	Dyad	Family	Com-munity	Society
(Clark, Riggle, Rostosky, Rothblum, & Balsam, 2015)	U.S. [2]	Qual	Mixed	Less than 25%				X	X		
(DiGregorio, 2016)	U.S. [3]	Qual	Women only	N/A or not measured	X			X			X
(Everett, Hatzenbuehler, & Hughes, 2016)	U.S. [2]	Quant	Women only	50% to 74%	X	X	X				
(Flores & Barclay, 2016)	U.S. [1]	Quant	N/A or not measured	N/A or not measured							X
(Gash & Raiskin, 2018)	U.S. [2]	Qual	Mixed	N/A or not measured							X
(Goodwin & Butler, 2009)	Europe	Qual	Mixed	Less than 25%				X			
(Haas & Whitton, 2015)	U.S. [1]	Qual	Mixed & TG/GNB included	Less than 25%				X			
(Hooghe & Meeusen, 2013)	Europe	Quant	N/A or not measured	N/A or not measured							X
(Hull, 2019)	U.S. [2]	Qual	Mixed & TG/GNB included	25 to 49%			X				
(Jowett & Peel, 2017)	Europe	Qual	Mixed & TG/GNB included	Less than 25%			X				
(Kazyak & Stange, 2018)	U.S. [2]	Quant	Mixed	N/A or not measured							X
(Kennedy, Dalla, & Dreesman, 2018)	U.S. [3]	Mixed	Mixed & TG/GNB included	Less than 25%					X	X	

Citation	Location	Methods	Participant gender(s)	Percent people of color	SMW-specific	Race/ ethnicity-specific	Indivi-dual	Dyad	Family	Com-munity	Society
(Kreitzer, Hamilton, & Tolbert, 2014)	U.S. [2]	Quant	N/A or not measured	N/A or not measured							X
(Lannutti, 2005)	U.S. [2]	Qual	Mixed & TG/GNB included	Less than 25%			X			X	
(Lannutti, 2007b)	U.S. [2]	Qual	Women only	25 to 49%	X				X	X	
(Lannutti, 2007a)	U.S. [2]	Qual	Mixed & TG/GNB included	Less than 25%			X	X			
(Lannutti, 2008)	U.S. [2]	Qual	Mixed	Less than 25%					X		
(Lannutti, 2011a)	U.S. [3]	Qual	Mixed	Less than 25%						X	
(Lannutti, 2011b)	U.S. [2]	Qual	Mixed	Less than 25%			X	X	X	X	
(Lannutti, 2013)	U.S. [2]	Qual	Mixed	25 to 49%					X		
(Lannutti, 2018a)	U.S. [3]	Qual	Mixed	Less than 25%					X	X	
(Lannutti, 2018b)	U.S. [3]	Qual	Mixed	25 to 49%					X		
(LeBlanc, Frost, & Bowen, 2018)	U.S. [1]	Quant	Mixed	25 to 49%	X			X			
(Lee, 2018)	U.S. [1]	Quant	Mixed	75%+	X	X	X				
(MacIntosh, Reissing, & Andruff, 2010)	Canada	Mixed	Mixed	N/A or not measured				X			
(Maisel & Fingerhut, 2011)	U.S. [2]	Quant	Mixed	Less than 25%	X					X	
(McGuffey, 2018)	U.S. [3]	Qual	Mixed & TG/GNB included	75%+		X	X				
(Metheny & Stephenson, 2019)	U.S. [1]	Quant	Men only	25 to 49%		X	X				

Citation	Location	Methods	Participant gender(s)	Percent people of color	SMW-specific	Race/ ethnicity-specific	Indivi-dual	Dyad	Family	Com-munity	Society
(Ocobock, 2013)	U.S. [2]	Qual	Men only	Less than 25%					X		
(Ocobock, 2018)	U.S. [2]	Mixed	Mixed	Less than 25%			X			X	
(Ofosu, Chambers, Chen, & Hehman, 2019)	U.S. [1]	Quant	Mixed	N/A or not measured							X
(Ogolsky, Monk, Rice, & Oswald, 2019)	U.S. [1]	Quant	Mixed & TG/GNB included	Less than 25%					X		
(Perrin, Smith, Trujillo, Rabinovitch, & Coy, 2018)	U.S. [1]	Quant	N/A or not measured	N/A or not measured							X
(Philpot et al., 2016)	Australia /NZ	Mixed	Men only	N/A or not measured			X				
(Redman, 2018)	Europe	Quant	N/A or not measured	N/A or not measured							X
(Reynolds & Robinson, 2019)	Australia /NZ	Qual	Mixed	N/A or not measured			X				
(Riggle, Wickham, Rostosky, Rothblum, & Balsam, 2017)	U.S. [3]	Quant	Mixed	Less than 25%			X		X		
(Riggle, Drabble, Veldhuis, Wootton, & Hughes, 2018)	U.S. [3]	Qual	Women only	50% to 74%	X				X		
(Rostosky, Riggle, Rothblum, & Balsam, 2016)	U.S. [3]	Qual	Mixed	Less than 25%				X	X		
(Sansone, 2019)	U.S. [1]	Quant	Mixed	N/A or not measured							X

Citation	Location	Methods	Participant gender(s)	Percent people of color	SMW-specific	Race/ ethnicity-specific	Indivi-dual	Dyad	Family	Com-munity	Society
(Schecter, Tracy, Page, & Luong, 2008)	U.S. [2]	Qual	Mixed	N/A or not measured					X		
(Scott & Theron, 2019)	Multiple countries	Qual	Women only	N/A or not measured	X			X			
(Shulman, Weck, Schwing, Smith, & Coale, 2009)	U.S. [2]	Qual	Mixed	Less than 25%				X			
(Shulman, Gotta, & Green, 2012)	U.S. [2]	Mixed	Mixed	Less than 25%				X			
(Tankard & Paluck, 2017)	U.S. [1]	Quant	N/A or not measured	N/A or not measured							X
(Thomas, 2014)	Multiple countries	Qual	Mixed	N/A or not measured				X	X		
(Vuckovic Juros, 2019)	Europe	Qual	Mixed	N/A or not measured					X		
(Wootton et al., 2019)	U.S. [3]	Qual	Women only	50% to 74%	X					X	

* *Note:* We endeavored to minimize redundancy in our summary of the literature by discussing articles in relation to the levels of the social ecological and stigma framework that aligned most closely with the study findings. However, findings of some manuscripts may be relevant to other social-ecological levels beyond the primary categorization used in the current scoping review.

[1] National U.S. sample; [2] Sample from single U.S. state; [3] Sample from multiple states; [4] Other – review of state and federal legal decisions

REFERENCES

Abou-Chadi, T., & Finnigan, R. (2019). Rights for same-sex couples and public attitudes toward gays and lesbians in Europe. *Comparative Political Studies, 52*(6), 868-895. doi:10.1177/0010414018797947

Aksoy, C. G., Carpenter, C. S., De Haas, R., & Tran, K. D. (2020). Do laws shape attitudes? Evidence from same-sex relationship recognition policies in Europe. *European Economic Review, 124*, 103399. doi:10.1016/j.euroecorev.2020.103399

Alderson, K. G. (2004). A phenomenological investigation of same-sex marriage. *Canadian Journal of Human Sexuality, 13*(2), 107-122.

Badgett, M. V. L. (2011). Social inclusion and the value of marriage equality in Massachusetts and the Netherlands. *Journal of Social Issues, 67*(2), 316-334. doi:10.1111/j.1540-4560.2011.01700.x

Balsam, K. F., Rostosky, S. S., & Riggle, E. D. B. (2017). Breaking up is hard to do: Women's experience of dissolving their same-sex relationship. *Journal of Lesbian Studies, 21*(1), 30-46. doi:10.1080/10894160.2016.1165561

Bernstein, M., Harvey, B., & Naples, N. A. (2018). Marriage, the final frontier? Same-sex marriage and the future of the lesbian and gay movement. *Sociological Forum, 33*(1), 30-52. doi:10.1111/socf.12392

Bernstein, M., Naples, N. A., & Harvey, B. (2016). The meaning of marriage to same-sex families: Formal partnership, parenthood, gender, and the welfare state in international perspective. *Social Politics, 23*(1), 3-39. doi:10.1093/sp/jxv002

Bishin, B. G., Hayes, T. J., Incantalupo, M. B., & Smith, C. A. (2016). Opinion backlash and public attitudes: Are political advances in gay rights counterproductive? *American Journal of Political Science, 60*(3), 625-648. doi:10.1111/ajps.12181

Bosley-Smith, E. R., & Reczek, C. (2018). Before and after 'I Do': Marriage processes for mid-life gay and lesbian married couples. *Journal of Homosexuality, 65*(14), 1985-2004. doi:10.1080/00918369.2017.1423213

Charlton, B. M., Corliss, H. L., Spiegelman, D., Williams, K., & Austin, S. B. (2016). Changes in reported sexual orientation following US states recognition of same-sex couples. *American Journal of Public Health, 106*(12), 2202-2204. doi:10.2105/AJPH.2016.303449

Chauveron, L. M., Alvarez, A., & van Eeden-Moorefield, B. (2017). The co-evolution of marriage and parental rights of gays and lesbians. *Journal of GLBT Family Studies, 13*(2), 114-136. doi:10.1080/1550428X.2016.1187105

Clark, J. B., Riggle, E. D. B., Rostosky, S. S., Rothblum, E. D., & Balsam, K. F. (2015). Windsor and Perry: Reactions of siblings in same-sex and heterosexual couples. *Journal of Homosexuality, 62*(8), 993-1008. doi:10.1080/00918369.2015.1039360

DiGregorio, N. (2016). Same-sex marriage policies and lesbian family life. *Sexuality Research & Social Policy: A Journal of the NSRC, 13*(1), 58-72. doi:10.1007/s13178-015-0211-z

Everett, B. G., Hatzenbuehler, M. L., & Hughes, T. L. (2016). The impact of civil union legislation on minority stress, depression, and hazardous drinking in a diverse sample of sexual-minority women: A quasi-natural experiment. *Social Science & Medicine, 169*, 180-190. doi:10.1016/j.socscimed.2016.09.036

Flores, A. R., & Barclay, S. (2016). Backlash, consensus, legitimacy, or polarization: The effect of same-sex marriage policy on mass attitudes. *Political Research Quarterly, 69*(1), 43-56. doi:10.1177/1065912915621175

Gash, A., & Raiskin, J. (2018). Parenting without protection: How legal status ambiguity affects lesbian and gay parenthood. *Law and Social Inquiry-Journal of the American Bar Foundation, 43*(1), 82-118. doi:10.1111/lsi.12233

Goodwin, C., & Butler, C. (2009). Legitimate love: The meaning of civil partnership for the positioning of lesbian and gay people in society. *Sexual and Relationship Therapy, 24*(3-4), 235-248. doi:10.1080/14681990903233061

Haas, S. M., & Whitton, S. W. (2015). The significance of living together and importance of marriage in same-sex couples. *Journal of Homosexuality, 62*(9), 1241-1263. doi:10.1080/00918369.2015.1037137

Hooghe, M., & Meeusen, C. (2013). Is same-sex marriage legislation related to attitudes toward homosexuality?: Trends in tolerance of homosexuality in European countries between 2002 and 2010. *Sexuality Research & Social Policy: A Journal of the NSRC, 10*(4), 258-268. doi:10.1007/s13178-013-0125-6

Hull, K. E. (2019). Same-sex marriage: Principle versus practice. *International Journal of Law Policy and the Family, 33*(1), 51-74. doi:10.1093/lawfam/eby018

Jowett, A., & Peel, E. (2017). 'A question of equality and choice': Same-sex couples' attitudes towards civil partnership after the introduction of same-sex marriage. *Psychology & Sexuality, 8*(1-2), 69-80. doi:10.1080/19419899.2017.1319408

Kazyak, E., & Stange, M. (2018). Backlash or a positive response?: Public opinion of LGB issues After Obergefell v. Hodges. *Journal of Homosexuality, 65*(14), 2028-2052. doi:10.1080/00918369.2017.1423216

Kennedy, H. R., Dalla, R. L., & Dreesman, S. (2018). "We are two of the lucky Ones": Experiences with marriage and wellbeing for aame-sex couples. *Journal of Homosexuality, 65*(9), 1207-1231. doi:10.1080/00918369.2017.1407612

Kreitzer, R. J., Hamilton, A. J., & Tolbert, C. J. (2014). Does policy adoption change opinions on minority rights? The effects of legalizing same-sex marriage. *Political Research Quarterly, 67*(4), 795-808.

Lannutti, P. J. (2005). For better or worse: Exploring the meanings of same-sex marriage within the lesbian, gay, bisexual and transgendered community. *Journal of Social and Personal Relationships, 22*(1), 5-18. doi:10.1177/0265407505049319

Lannutti, P. J. (2007a). The influence of same-sex marriage on the understanding of same-sex relationships. *Journal of Homosexuality, 53*(3), 135-151. doi:10.1300/J082v53n03_08

Lannutti, P. J. (2007b). 'This is not a lesbian wedding': Examining same-sex marriage and bisexual-lesbian couples. *Journal of Bisexuality, 7*(3-4), 237-260. doi:10.1080/15299710802171316

Lannutti, P. J. (2008). Attractions and obstacles while considering legally recognized same-sex marriage. *Journal of GLBT Family Studies, 4*(2), 245-264. doi:10.1080/15504280802096914

Lannutti, P. J. (2011a). Examining communication about marriage amendments: Same-sex couples and their extended social networks. *Journal of Social Issues, 67*(2), 264-281. doi:10.1111/j.1540-4560.2011.01697.x

Lannutti, P. J. (2011b). Security, recognition, and misgivings: Exploring older same-sex couples' experiences of legally recognized same-sex marriage. *Journal of Social and Personal Relationships, 28*(1), 64-82. doi:10.1177/0265407510386136

Lannutti, P. J. (2013). Same-sex marriage and privacy management: Examining couples' communication with family members. *Journal of Family Communication, 13*(1), 60-75. doi:10.1080/15267431.2012.742088

Lannutti, P. J. (2018a). Committed, unmarried same-sex couples and their social networks in the United States: Relationships and discursive strategies. *Journal of Homosexuality, 65*(9), 1232-1248. doi:10.1080/00918369.2017.1411690

Lannutti, P. J. (2018b). GLBTQ people who decided to marry after the 2016 US election: Reasons for and meanings of marriage. *Journal of GLBT Family Studies, 14*(1-2), 85-100. doi:10.1080/1550428X.2017.1420846

LeBlanc, A. J., Frost, D. M., & Bowen, K. (2018). Legal marriage, unequal recognition, and mental health among same-sex couples. *Journal of Marriage and Family, 80*(2), 397-408. doi:10.1111/jomf.12460

Lee, J. (2018). Black LGB identities and perceptions of same-sex marriage. *Journal of Homosexuality, 65*(14), 2005-2027. doi:10.1080/00918369.2017.1423214

MacIntosh, H., Reissing, E. D., & Andruff, H. (2010). Same-sex marriage in Canada: The impact of legal marriage on the first cohort of gay and lesbian Canadians to wed. *Canadian Journal of Human Sexuality, 19*(3), 79-90.

Maisel, N. C., & Fingerhut, A. W. (2011). California's ban on same-sex marriage: The campaign and its effects on gay, lesbian, and bisexual individuals. *Journal of Social Issues, 67*(2), 242-263. doi:10.1111/j.1540-4560.2011.01696.x

McGuffey, C. S. (2018). Intersectionality, cognition, disclosure and Black LGBT views on civil rights and marriage equality: Is gay the new Black? *Du Bois Review-Social Science Research on Race, 15*(2), 441-465. doi:10.1017/s1742058x18000218

Metheny, N., & Stephenson, R. (2019). Political environment and perceptions of social inclusion after nationwide marriage equality among partnered men who have sex with men in the USA. *Sexuality Research & Social Policy: A Journal of the NSRC, 16*(4), 521–528. doi:10.1007/s13178-018-0357-6

Ocobock, A. (2013). The power and limits of marriage: married gay men's family relationships. *Journal of Marriage and Family, 75*(1), 191-205. doi:10.1111/j.1741-3737.2012.01032.x

Ocobock, A. (2018). Status or access? The impact of marriage on lesbian, gay, bisexual, and queer community change. *Journal of Marriage and Family, 80*(2), 367-382. doi:10.1111/jomf.12468

Ofosu, E. K., Chambers, M. K., Chen, J. M., & Hehman, E. (2019). Same-sex marriage legalization associated with reduced implicit and explicit antigay bias. *PNAS Proceedings of the National Academy of Sciences of the United States of America, 116*(18), 8846-8851. doi:10.1073/pnas.1806000116

Ogolsky, B. G., Monk, J. K., Rice, T. M., & Oswald, R. F. (2019). Personal well-being across the transition to marriage equality: A longitudinal analysis. *Journal of Family Psychology, 33*(4), 422-432. doi:10.1037/fam0000504

Perrin, P. B., Smith, E. R., Trujillo, M. A., Rabinovitch, A., & Coy, A. E. (2018). Differential effects of the US Supreme Court's same-sex marriage decision on national support for lesbian, gay, and bisexual civil rights and sexual prejudice. *Sexuality Research & Social Policy, 15*(3), 342-352. doi:10.1007/s13178-017-0302-0

Philpot, S. P., Ellard, J., Duncan, D., Dowsett, G. W., Bavinton, B. R., Down, I., . . . Prestage, G. (2016). Gay and bisexual men's interest in marriage: An Australian perspective. *Culture, Health & Sexuality, 18*(12), 1347-1362. doi:10.1080/13691058.2016.1184314

Redman, S. M. (2018). Effects of same-sex legislation on attitudes toward homosexuality. *Political Research Quarterly, 71*(3), 628-641. doi:10.1177/1065912917753077

Reynolds, R., & Robinson, S. (2019). Marriage as a marker of secular inclusion? Oral history and lesbian and gay narratives on marriage in contemporary Australia. *Journal of Religious History, 43*(2), 269-284. doi:10.1111/1467-9809.12591

Riggle, E. D. B., Drabble, L., Veldhuis, C., Wootton, A., & Hughes, T. L. (2018). The impact of marriage equality on sexual minority women's relationships with their families of origin. *Journal of Homosexuality, 65*(9), 1190-1206. doi:10.1080/00918369.2017.1407611

Riggle, E. D. B., Wickham, R. E., Rostosky, S. S., Rothblum, E. D., & Balsam, K. F. (2017). Impact of civil marriage recognition for long-term same-sex couples. *Sexuality Research & Social Policy, 14*(2), 223-232. doi:10.1007/s13178-016-0243-z

Rostosky, S. S., Riggle, E. D. B., Rothblum, E. D., & Balsam, K. F. (2016). Same-sex couples' decisions and experiences of marriage in the context of minority stress: Interviews from a population-based longitudinal study. *Journal of Homosexuality, 63*(8), 1019-1040. doi:10.1080/00918369.2016.1191232

Sansone, D. (2019). Pink work: Same-sex marriage, employment and discrimination. *Journal of Public Economics, 180*, 20. doi:10.1016/j.jpubeco.2019.104086

Schecter, E., Tracy, A. J., Page, K. V., & Luong, G. (2008). Shall we marry? Legal marriage as a commitment event in same-sex relationships. *Journal of Homosexuality, 54*(4), 400-422. doi:10.1080/00918360801991422

Scott, J., & Theron, L. (2019). The promise of heteronormativity: Marriage as a strategy for respectability in South Africa. *Sexualities, 22*(3), 436-451. doi:10.1177/1363460717713384

Shulman, J. L., Gotta, G., & Green, R.-J. (2012). Will marriage matter? Effects of marriage anticipated by same-sex couples. *Journal of Family Issues, 33*(2), 158-181. doi:10.1177/0192513X11406228

Shulman, J. L., Weck, V., Schwing, S., Smith, T., & Coale, E. (2009). The push-pull of policy pressure: A qualitative exploration of the experiences of same-sex marriage policies among non-metropolitan GLB individuals. *Journal of GLBT Family Studies, 5*(4), 340-365. doi:10.1080/15504280903263785

Tankard, M. E., & Paluck, E. L. (2017). The effect of a Supreme Court decision regarding gay marriage on social norms and personal attitudes. *Psychological Science, 28*(9), 1334-1344. doi:10.1177/0956797617709594

Thomas, M. (2014). Atrocity stories and triumph stories: Using couple narratives to evaluate same-sex marriage and civil partnership. *Narrative Inquiry, 24*(2), 200-217. doi:10.1075/ni.24.2.02tho

Vuckovic Juros, T. (2019). Transformative power of same-sex marriage and non-heterosexual reproductivity How parents of glb offspring adjust to their marriage and children. *Journal of GLBT Family Studies*. doi:10.1080/1550428X.2019.1650407

Wootton, A. R., Drabble, L. A., Riggle, E. D. B., Veldhuis, C. B., Bitcon, C., Trocki, K. F., & Hughes, T. L. (2019). Impacts of marriage legalization on the experiences of sexual minority women in work and community contexts. *Journal of GLBT Family Studies, 15*(3), 211-234 doi:10.1080/1550428X.2018.1474829
