# Supplementary material for: Perceived psychosocial impacts of legalized same-sex marriage: A scoping review of sexual minority adults’ experiences
Source: PLoS One. 2021 May 6;16(5):e0249125. doi: 10.1371/journal.pone.0249125 (PMC8101749; doi:10.1371/journal.pone.0249125)
Supplement: S1 Text — (DOCX) [file pone.0249125.s003.docx]

**S1 Text. Definitions.**

Sexual minority: Individuals whose sexual identity, behavior, or attraction differ from the majority of the surrounding society, including lesbian, gay, bisexual, and queer identified individuals as well as those individuals who endorse other identities that are not exclusively heterosexual.

Sex: Differences between women and men are referred to as sex differences.

Gender: Gender refers to socially constructed and enacted roles and behaviors and includes gender identity (how individuals or groups perceive themselves) and gender norms.

Gender minority: Individuals whose gender identity or gender expression do not conform to traditional notions of binary gender typically associated with sex assigned at birth.

Binary Gender: Social construction of only two, rigid gender categories, male/man and female/woman, and corresponding cultural expectations of masculine and feminine expression and roles.

Same-sex marriage: Legally recognized civil marriage of two people of the same sex.

Equal marriage rights: The right to marry; terms and conditions of marriage are equal for all couples (regardless of the sex/gender of the partners).

Obergefell: Refers to the U.S. Supreme Court decision in Obergefell v. Hodges 576 U.S., released June 25, 2015, which extended the marriage rights to same-sex couples in all U.S. states.

LGBTQ+ community: An inclusive term used to refer to groups of sexual and gender minority people (e.g., lesbian, gay, bisexual, transgender, queer, questioning, etc.), such as LGBTQ+ social groups and activist groups.

Local community contexts: Local geographic and organization contexts in which individuals interact with others, including local residential communities, workplaces, schools, and religious organizations.

Extended social networks: Includes individuals that people associate with but are not particularly close to, such as acquaintances, neighbors, and friends of friends.
